# Palaeoproteomic investigation of archaeological sediments associated with ochre use in the Middle Stone Age of southern Africa

**DOI:** 10.1098/rspb.2025.3300

**Published:** 2026-10-21

**Authors:** Zahra Haghighi, Takumi Tsutaya, Elizabeth C. Velliky, Simon Armitage, Christopher Miller, Meaghan Mackie, Francesco d'Errico, Gaudry Troche, Jesper Olsen, Karen van Niekerk, Christopher Henshilwood, Enrico Cappellini

**Affiliations:** ^1^ SFF Centre for Early Sapiens Behaviour (SapienCE), University of Bergen, 5007 Bergen, Norway; ^2^ Research Center for Integrative Evolutionary Science, The Graduate University for Advanced Studies (SOKENDAI), Hayama, Kanagawa 240-0193, Japan; ^3^ Institute for Advanced Study, Kyushu University, Fukuoka 819-0395, Japan; ^4^ Globe Institute, University of Copenhagen, 1350 Copenhagen K, Denmark; ^5^ Interdisciplinary Center for Archaeology and Evolution of Human Behaviour (ICArEHB), University of Algarve, 8005-139 Faro, Portugal; ^6^ Department of Geography, Royal Holloway, University of London, Egham, Surrey TW20 0EX, UK; ^7^ Institute for Archaeological Sciences and Senckenberg Centre for Human Evolution and Palaeoenvironment, University of Tübingen, 72074 Tübingen, Germany; ^8^ Novo Nordisk Foundation Center for Protein Research, University of Copenhagen, 2200 Copenhagen, Denmark; ^9^ CNRS UMR5199 PACEA, Université de Bordeaux, 33615 Pessac Cedex, France; ^10^ School of Geography, Archaeology and Environmental Studies, University of the Witwatersrand, Johannesburg 2000, South Africa; ^11^ Evolutionary Studies Institute, University of the Witwatersrand, Johannesburg 2000, South Africa

**Keywords:** ancient protein residues, palaeoproteomics, common eland, Howiesons Poort, Klipdrift Shelter, African Middle Stone Age, South Africa

## Abstract

The African Middle Stone Age (MSA) represents the cultural and technological context associated with the emergence of *Homo sapiens* and the development of complex cognition. In this setting, ochre use is considered a multifaceted proxy for modern behaviour, allowing inferences about technical and symbolic aspects of *Homo sapiens*. Powdered ochre is reported in multiple southern African ethnographic and archaeological contexts alongside various organic materials, some of which are protein-based. Yet, the recovery of proteinaceous residues is rare in the MSA. Since proteins can offer insights into their tissue and species of origin, identifying proteins in association with ochre may help to infer its function. At Klipdrift Shelter (South Africa), the 60 000-year-old Howiesons Poort layers contain a laterally continuous, ochre-rich sediment surface, possibly over 1 m long, 40 cm wide, and up to 1 cm thick. We used palaeoproteomics to identify proteinaceous traces from this feature. Our analysis identified type I collagen confidently matching common eland (*Taurotragus oryx*) in two samples. Together with the feature's highly complex nature, these findings suggest the deliberate mixing of eland-derived proteins with ochre powder. Our results confirm that palaeoproteomics can be a reliable tool for reconstructing the protein component of the early human material culture.

## 1. Introduction

The African Middle Stone Age (MSA) represents a pivotal period in human prehistory, characterized by the emergence of *Homo sapiens* and the subsequent introduction of several key cultural and technological innovations. These cognitively demanding innovations include the use of mineral pigments such as iron oxides, commonly referred to as ochre [1–3], sophisticated tool production methods [4,5], the use of personal ornaments [6,7] and organic and inorganic materials to produce compounds [8], usually for the hafting of tools [9,10]. These activities often require multitasking, planning and knowledge of the surrounding environment. This implies the need to transmit a large and complex body of knowledge to the new generation, potentially leading to multi-generation accumulation of technical expertise [11–13].

The geographically widespread, intensive and habitual use of ochre, a natural earth pigment [14,15], in the southern African MSA has long been interpreted to be a symbolically mediated behaviour [16]. For example, the incised ochre pieces dating back to 75–100 thousand years ago (ka) from Blombos Cave (BBC), South Africa, were arguably created with symbolic intent [1,17]. At most MSA sites, evidence of anthropogenic extraction of the ochre pigment, such as scraping and grinding marks, is frequently encountered [13,18–20]. Pigment residues are often found on artefacts such as grindstones, beads and shell pieces. At Blombos Cave, for instance, two abalone shells containing an ochre-rich compound dating back to around 100 ka were discovered [8]. The association of burnt and crushed bone with one of these shells suggests that powdered ochre was likely mixed with substances, such as fat and bone marrow, to produce paint-like mixtures [8]. Additionally, at Sibudu, a mixture of ochre and milk (approx. 49 ka) was identified as a paint medium [21]. These mixtures were probably used for decorative or symbolic purposes, such as body painting [16,22]. Studies from both the MSA and Later Stone Age (LSA) finds demonstrate that ochre is an effective loading agent for hafting [23], with some evidence of powdered ochre being mixed with various organic substances, such as plant gum, animal fat, beeswax and proteinaceous materials [24–26], to manufacture compound adhesives for attaching stone tips to handles or shafts [9,27,28]. Alternatively, red ochre may have been employed for hide processing, such as for colouring or ‘loading’ [29], for mechanical finishing or cleaning [30] or for preventing decomposition of hides, and making them softer and impermeable [31]. Although there is no direct evidence for hide working during the MSA, several findings suggest that it occurred. Evidence includes several ochre-stained bone tools in BBC (approx. 77 ka), interpreted as awls for piercing hides [5], lithic scrapers with a range of micro-residues, consisting of ochre–fat mixture, muscle tissues and plant gum (approx. 58 ka) from Sibudu [32] and rubbed ochre in Rose Cottage MSA deposits [18]. In particular, the discovery of leather clothing in LSA deposits at several southern African archaeological sites [33–35] suggests that this practice was relatively common during this period and may have origins in earlier times. Other potential functional applications of ochre include sunscreen [36] and medication [37].

In addition to the well documented ochre occurrences discussed above, microscopic ochre fragments have also been found as localized patches dispersed within sediments. These features are observed as thin, ochre-rich surfaces during excavation, but are rarely sampled *in situ* and examined. At Sibudu, abundant powdered ochre deposits were discovered on cemented white ash of hearths, dating to about 58 ka. These cemented ashes may have served as either receptacles for powdered ochre or work surfaces for ochre grinding on coarse slabs [38]. Continuous ochre-rich layers have also been documented at Klipdrift Shelter (KDS) [39] and Blombos Cave (see fig. 2 from [8] and figs. 17–19 from [40]). Both of these contexts represent laterally continuous, less than 1 cm thick powdered ochre deposits. Although from a younger and different context, at La Prele Mammoth Site (13 ka) in the USA, a large ochre surface associated with a hearth was interpreted as a domestic space, because the authors were able to rule out a geogenic (formed through natural geological processes) origin. Possibly, this surface was used for treating hide, since La Prele is considered a mammoth processing site [41]. At the European Palaeolithic site of Geißenklösterle Cave in southwestern Germany, an ochre surface was documented in the Aurignacian layers. The surface was initially interpreted as an ochre-covered animal hide owing to its oily texture [42]. However, a later study suggested that the ochre staining resulted from post-depositional iron leaching through the sediments, rather than anthropogenic activities. This reinterpretation, however, was based solely on soil-sample analysis and did not involve micromorphological investigation [43]. Additionally, ochre has been found in burials and as possible ‘floor’ deposits in various sites across Europe and the Levant [44–47]. These deposits probably reflect a variety of practices, such as ochre mixtures applied to animal skins or objects, or as part of burial rituals. Taken together, these examples underscore the complexity of ochre-related behaviours and highlight the importance of further studies on *in situ* deposits or samples to gain deeper insights into its diverse functions in ancient contexts. Notably, in the majority of examples discussed above, powdered ochre residues have often been associated with various organic components, including plant-based materials (e.g. plant resins) and animal-derived products (e.g. blood, fat, milk and hide).

Our current understanding of the use of organic materials during the MSA (and LSA) is largely informed by ethnographic accounts and relatively extensive studies of visible adhesive residues on tools [9,10,25,26,48,49]. Beyond adhesives, organic compounds are generally understudied, likely because of their degradation and owing to the absence of visible traces in the archaeological record. Notably, among biogenic materials, humans have often been using protein-based ones for multiple purposes. The molecular detection of these proteins can reveal precious information about their function or their tissue and species of origin. This information can then be used to infer broader archaeological questions, such as past subsistence strategies, symbolic behaviour and social organization. Identifying specific protein residues associated with MSA materials may thus be a key step toward gaining a deeper insight into the biological component of early human material culture.

Several analytical techniques, such as gas chromatography coupled to mass spectrometry (GC–MS), have enabled the broad detection of protein traces along with other organic compounds, including lipids, waxes, resins and polysaccharides [21,26,49,50]. However, these methods often lack the specificity and sensitivity required to reconstruct the amino acid sequence of degraded protein fragments. By contrast, palaeoproteomics, through the use of liquid chromatography coupled to tandem mass spectrometry (LC–MS/MS), enables accurate protein identification, allowing more precise inferences about the biological sources of these residues.

Ancient proteins have proven more resilient than ancient DNA (aDNA), with a greater capacity to survive over extended time spans and under harsher, warmer environmental conditions [51–54]. In African contexts, recent studies have demonstrated that aDNA preservation is generally poor, with the oldest DNA recovered dating back to approximately 18 ka [55], during the LSA period (approx. 40–500 ka [56–58]). The molecular characterization of ancient protein traces may thus represent the sole viable option to confidently identify the use of specific materials of biological origin in the MSA (approx. 250–40 ka [59]) archaeological contexts.

To date, successful applications of palaeoproteomics on southern African archaeological materials have primarily focused on recovering collagen proteins from mineralized tissues, such as bone and ivory artefacts, particularly from more recent contexts. These studies aim to identify animal species in order to shed light on various historical activities, including the ivory trade (7th–10th centuries AD) [60], the manufacture of bone arrowheads (4th–7th centuries AD) [61] and early pastoralism [62,63]. There are, however, only two studies that have utilized palaeoproteomics to investigate older materials. Nel *et al.* studied micromammal bone remains from the MSA using palaeoproteomics to reconstruct the environments that existed in KDS, South Africa during the deposition of the Howiesons Poort (HP) levels [64]. Additionally, by using palaeoproteomics, Villa *et al.* identified caseins from ochre mixture residue on a stone flake (approx. 49 ka) from Sibudu, South Africa [21]. Accordingly, the potential of palaeoproteomics to unlock otherwise unattainable information in African contexts, particularly within the MSA, remains largely unexplored.

We used palaeoproteomics to investigate potential protein residues preserved within samples collected from the ochre-rich sediment surface at KDS. By identifying any traces of proteins, we aimed to shed light on the potential processes that contributed to the formation and the subsequent decomposition of this distinctive feature, whether resulting from human activities or of geogenic origin. Furthermore, this study sought to better understand the purpose behind the use or presence of ochre in this context, exploring whether it served functional, symbolic or ritual roles. Ultimately, this study aimed to better understand the cultural practices of early populations of the southern African hunter–gatherers, as well as their interactions with, and manipulations of, their environment, through the new insights provided by ancient protein analysis.

## 2. Material and methods

### (a) The Middle Stone Age ochre-rich sediment feature at Klipdrift Shelter

KDS was interpreted as an occupation site at which a variety of activities, such as ochre processing, fire making, skinning, and marrow extraction, took place [65]. The site (34°27.09630ʹ S, 20°43.45820ʹ E) was located approximately 150 km east of Cape Town in the De Hoop Nature Reserve, South Africa [66], and was part of the Klipdrift Complex (supplementary material, figures S1 and S2), which also included the Klipdrift Cave (KDC) and the Klipdrift Cave Lower (KDCL). KDS was organized into a square-metre grid system (figure 1*a,b*; supplementary material, figure S2), with each square subdivided into four quadrants (a, b, c and d). The stratigraphic layers (PBD, PBE, PCA and PBA–PBB in this study) were ordered alphabetically, starting from the uppermost layer and progressing to the lowermost, with layers sharing the same first two letters indicating close contextual relationships [66].

**Figure 1.**
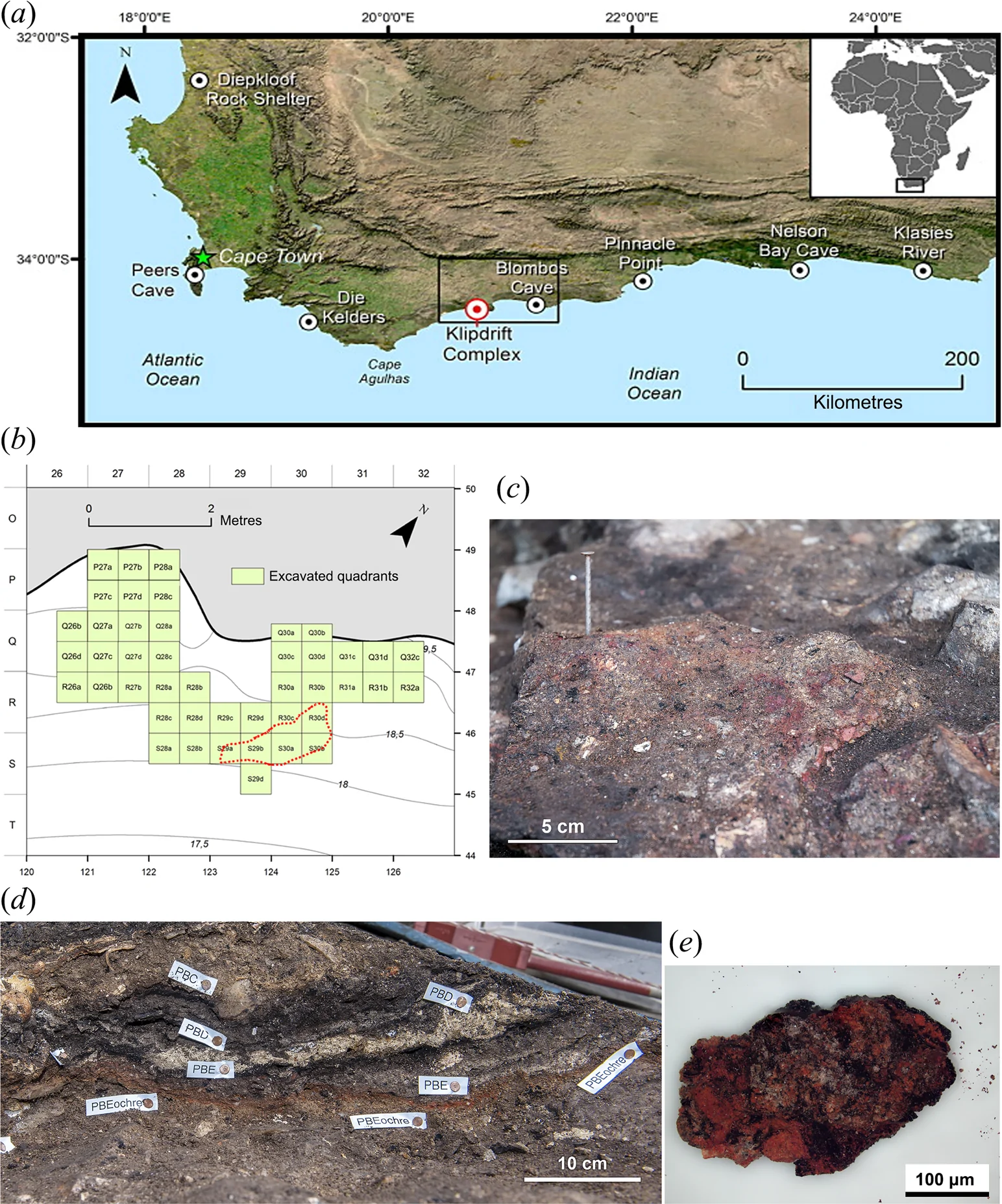
Overview of the ochre surface in layer PBE at Klipdrift Shelter. (*a*) Map showing the location of the Klipdrift archaeological sites in South Africa. (*b*) Planar view of the potential outline of the ochre surface in layer PBE, with (*c*) a close-up view of the sediment in the surface and (*d*) a profile view during excavation. (*e*) Microscopic image of an ochre-rich sediment sample. Images (*a*–*d*) courtesy of Magnus M. Haaland and Ole F. Unhammer.

KDS contained MSA deposits in 23 archaeostratigraphic units, dated to between approximately 52 ka (the uppermost layers) and approximately 72 ka (the lowest layers), by single-grain optically stimulated luminescence (OSL) methods (supplementary material, figure S2) [66]. Excavations started in 2011 and continued in 2012, 2013 and 2018, leading to the discovery of diverse cultural and faunal materials, including lithics, marine shells, terrestrial fauna, hearths, ochre and engraved ostrich eggshell [66]. A recent analysis of the ochre assemblage recovered during the 2011–2013 and 2018 excavation seasons identified a total of 597 pieces (supplementary material, table S2). The presence of multiple archaeological attributes strongly indicated that ochre processing was established and regularly practised during the Howiesons Poort at KDS [39]. This is further explained in the supplementary material, ‘Ochre occurrences at Middle Stone Age deposits of KDS’. Particularly, numerous patches of ochre powder were recovered throughout the HP layers at KDS in different sizes, textures (rounded, sub-rounded and sub-angular) and conglomerations. A unique feature associated with layer PBE was a laterally continuous ochre surface (figure 1*b-e*). Even though smaller patches of ochre were found throughout the HP layers, the size of the ochre layer in PBE was exceptional. The full extent of the ochre surface was now impossible to reconstruct as it was completely excavated during the 2011–2013 seasons, and its precise extent was not documented. It is possible that the smaller ochre patches found in other quadrants of layer PBE might represent fragments of this continuous surface. Thus, based on the photographs and site notes alone, it was possible that the ochre surface was over 1 m in length and 40 cm wide (figure 1*b-e*), while other small patches recovered from the other layers might represent remnants of similar surfaces. The surviving pieces showed thicknesses of roughly 5–7 mm, and it was possible that this feature reached approximately 1 cm in some areas.

For proteomic analysis, five archived sediment samples (supplementary material, figure S5) originating from different HP layers (specifically, layers PBD, PBE, PCA and PBA–PBB) were subsampled (approx. 30–150 mg each). These samples were originally collected during the 2011–2013 excavation seasons from the ochre-rich sediment surface feature. After collection, they were archived and stored at the University of Witwatersrand satellite laboratory in Cape Town. A summary of the samples is provided in supplementary material, table S2.

### (b) Palaeoproteomics

Protein residues in sediments might survive through mineral binding to various soil components or encapsulation within humic substances [67–70]. Previous research had demonstrated that conventional protein extraction methods for biomineralized tissues (e.g. bone) were not effective in recovering a substantial quantity of proteins from sediment samples [71]. The digestion-free method, initially designed to improve the recovery of randomly fragmented peptides from highly degraded proteins [72,73], was therefore used to identify ancient peptides from ochre-rich sediments. However, matching MS/MS spectra of randomly cleaved peptides against large reference databases was computationally unsustainable, owing to the explosion of the search space in this type of search. For this reason, we combined two different protein extraction conditions. First, we developed a new method, named AB (acid and base) digestion, that performed stepwise protein extraction and protease digestion under different denaturing conditions. This involved sequential treatment with acidic and basic solutions, followed by digestion with trypsin (visualized in supplementary material, figure S6). The AB-digestion method enabled profiling of the metaproteome composition of a sample using a large database (e.g. Swiss-Prot). This initial analysis provided a preliminary overview of the type of protein present in the sediment samples, which in this study was mammalian collagen. Based on this, a small, focused reference database (comprising mammalian collagen alpha-1 type I (COL1A1) and collagen alpha-2 type I (COL1A2) sequences) was constructed. Subsequently, the digestion-free method, which was more effective at extracting peptides from sediment, was applied. The resulting data were then searched against the restricted database. This two-step approach thus allowed more efficient identification of proteins present in the sediment than did the pure digestion-free method.

For the AB-digestion method, approximately 130 mg of two ochre-rich sediment samples (PBD_S28b and PBE_S29b) were transferred to Eppendorf Protein Lo-Bind tubes, denatured and incubated at 99°C for 10 min in 300 µl GuHCl buffer: 2 M guanidinium hydrochloride solution (GuHCl; Sigma-Aldrich, Germany) with 10 mM Tris(2-carboxyethyl)phosphine hydrochloride (TCEP; Sigma-Aldrich, Germany) and 20 mM chloroacetamide (CAA; Sigma-Aldrich, Germany), buffered with 100 mM Tris (Invitrogen, Germany). Then, 10 µl of 36% hydrochloric acid (HCl; Sigma-Aldrich, Germany) was added to bring the pH to less than 1.5. The mixture was then incubated at 4°C overnight to facilitate the cleavage of the bond between minerals and peptides. After the incubation, the samples were centrifuged (10 000*g*, 5 min), and the supernatant was collected as Supernatant 1 (S1). The pellet samples were washed twice with 300 µl 100 mM Tris solution in order to neutralize them. The washed Tris solution was collected and combined with S1. Then, 300 µl GuHCl buffer was added to the pellet fraction and incubated at 99°C for 10 min. After incubation, 3.0 M sodium hydroxide (NaOH; Sigma-Aldrich, Germany) was added to increase the pH above 13, and the mixture was incubated at room temperature for 20 min to remove the humic substances. The samples were centrifuged (10 000*g*, 5 min), and the supernatant was collected as Supernatant 2 (S2). The pellet samples were neutralized with two washes of 300 µl 100 mM Tris solution. The washed Tris solution was collected and combined with S2. Then, 300 µl GuHCl buffer was added to the pellet fraction and incubated at 99°C for 10 min, and the resultant fraction Pellet (P) was retained. The pH of S1, S2 and P fractions was adjusted to 7–9 with either 10 M HCl or 3.0 M NaOH. Then, all fractions underwent protein digestion under agitation at 37°C in-solution, first with 0.2 µg rLys-C (Promega, Sweden) for 2 h, before being diluted to a final concentration of 0.6 M GuHCl using 25 mM Tris in 10% acetonitrile (ACN, Thermo Fisher Scientific, Germany) in water and digested with 0.8 µg trypsin (Promega, Sweden) overnight. After trypsin digestion, the fractions were acidified to pH <2.0 using 10% trifluoroacetic acid (TFA; Merck, Germany) to terminate the digestion.

Afterwards, all samples (30–150 mg each) were treated with strong acid (10% TFA or HCl) and purified without protease digestion (‘digestion-free method’, see supplementary material) [72,73]. All fractions were treated along with experimental blanks. For both experiments, the extracted peptides were purified with C18 StageTips [74] and sequenced by LC-MS/MS (EASY-nLC 1200 and Exploris 480 Orbitrap mass spectrometer). The resultant mass spectrometry data were searched against the Swiss-Prot protein database (approx. 568 000 protein entries, downloaded 18 March 2022) using MaxQuant software (v. 2.6.3.0) with specific trypsin cleavages for the first experiment. Then, all mass spectrometry data, resulting from both extraction methods, were searched against a custom protein database with nonspecific protease cleavages. This database contained both 711 mammalian complete or partial COL1A1 and COL1A2 sequences registered in the UniProt database, as well as mature COL1A1 and COL1A2 sequences from 44 bovid species naturally distributed in Africa, initially generated by [75] and later updated by [63]. Software parameters common between the runs were set as follows: carbamidomethylation (C) was set as a fixed modification (for digested fractions), while deamidation (NQ), oxidation (M), hydroxylation (P), and cyclization of N-terminal glutamic acid (Glu) or glutamine (Gln) to pyroglutamate (pyro-Glu) were set as variable modifications. Minimum peptide length was set to 7. Protein and peptide identifications were filtered by a false discovery rate (FDR) of 0.01. The results of the final search were used for the subsequent data analysis using R software [76]. The deamidation rates of the identified proteins were calculated using an R script (rDeamidation) [77] modified from a Python script published by Mackie *et al.* [78]. Detailed methodology for protein extraction, mass spectrometry analysis and data analysis is available in the supplementary material. All mass spectrometry proteomics data, as well as the FASTA files, have been deposited in the ProteomeXchange Consortium (http://proteomecentral.proteomexchange.org) via the PRIDE partner repository [79] with the dataset identifier PXD069507.

### (c) Microscopic analysis

A total of 15 archived samples were collected and examined. Most of these came from the PBD and PBE layers (11 in total: 6 from PBD and 5 from PBE), with the remaining from PCA, PBA/PBB, PAV and PAU. The samples selected for microscopic analysis largely overlapped with those chosen for proteomics, being either subsampled directly from the same material used for proteomics analysis, or collected from the closest possible adjacent area, such as the same quadrant or a neighbouring one. Microscopic analyses of selected samples were carried out to determine whether bone fragments were present in the ochre-rich sediments, and if so, to determine their abundance. This analysis was necessary to determine the likelihood that potential proteins associated with ochre-rich material originated from the ochre sediments themselves, or from the bone fragments.

Initially, two different optical microscopes were used, with photographs being taken where necessary. The majority of the photographs were taken using a Nikon Eclipse Ci POL microscope paired with a Nikon DS-Fi3, using the following two objectives: Plan Fluor 4× OFN25 and Plan Fluor 10× OFN25 Ph1 DL. The few remaining photographs were taken by means of a Leica M205A microscope paired with a Leica EC3 digital colour camera.

Afterwards, a total of nine samples were investigated further using electron microscopy, employing both the specimen's microstructure and its elemental compositions as criteria for identification of bone fragments. Sediment samples were mounted onto an aluminium holder using carbon tape. A thin coating of carbon was applied using an Agar Turbo Carbon Coater, and in some cases, an additional coating of palladium was added using a Polaron Sputter Coater SC502, to ensure electrical conductivity. Samples intended for thin-section preparation were first stabilized through vacuum impregnation with epoxy resin to preserve their internal microstructures. Once stabilized and mounted onto glass slides, the sections were ground to a final thickness of 120 µm.

A Zeiss Supra 55VP scanning electron microscope (SEM) equipped with a Thermo Scientific Noran System 7 energy-dispersive X-ray spectroscopy (EDS) system was used to investigate the surface structure of the bone-like fragments as well as their elemental composition. For both samples (thin-sectioned and loose fragments), the imaging was performed with a secondary electron (SE) detector at 5 kV and 4 mm working distance and EDS maps and point analyses at 15 kV and 8.5 mm working distance. Because of the rough surface of the loose fragments, the EDS results from these samples were considered qualitative to semiquantitative. Further explanation can be found in supplementary material, ‘Microscopic analysis’. A visual representation of the approximate locations of ochre-rich sediment samples collected for proteomics and microscopy analyses is provided in supplementary material, figure S12.

## 3. Results

### (a) Palaeoproteomics

Collagen alpha-1 type I (COL1A1) and collagen alpha-2 type I (COL1A2) were confidently identified from two of the five ochre-rich sediment samples of KDS, originating from two different Howiesons Poort layers (PBD and PBE). Although the retrieved sequence of COL1A1 matched several African bovid species (supplementary material, figures S9 and S10), the combination of sequence fragments of COL1A2 exclusively matched the common eland (*Taurotragus oryx*), a large antelope native to southern Africa. A total of 69 razor + unique peptides, comprising 171 sequenced COL1A2 amino acid (aa) residues (i.e. 16.5% of the entire COL1A2 protein), were identified across five fractions of PBD and PBE sediment samples (supplementary material, tables S3–S7). The average depth of the peptide coverage was 4.1× for the identified 171 residues (supplementary material, table S5). The bootstrapped values of the deamidation rates of asparagine (N) and glutamine (Q) residues of COL1A1 and COL1A2 were mostly 100%, except for a few fractions with a relatively smaller number of identified peptides (supplementary material, tables S10 and S11). The peptide length distribution of COL1A1 and COL1A2 (from undigested fractions) has a maximum frequency at 17 aa residues (supplementary material, figures S7 and S8). A summary of the count of amino acids from the retrieved sequences of both COL1A1 and COL1A2 is provided in supplementary material, table S8.

The reconstructed peptide sequence from all fractions with and without protease digestion exclusively matched with the COL1A2 sequence of common eland. No sequence variants compatible with other species were observed. We confidently identified several amino acid variants that support parsimoniously assigning the origin of the identified collagens in the KDS ochre-rich sediment samples to *T. oryx*. Notably, COL1A2 with proline (P) in position 693, serine (S) in position 720 and threonine (T) in position 878 are only found in eland (figure 2; supplementary material, figure S11). The depth of coverage of these positions, for both 720S and 878T, with confident MS2 spectra is 6 and for 693P is 1 (figure 2).

**Figure 2.**
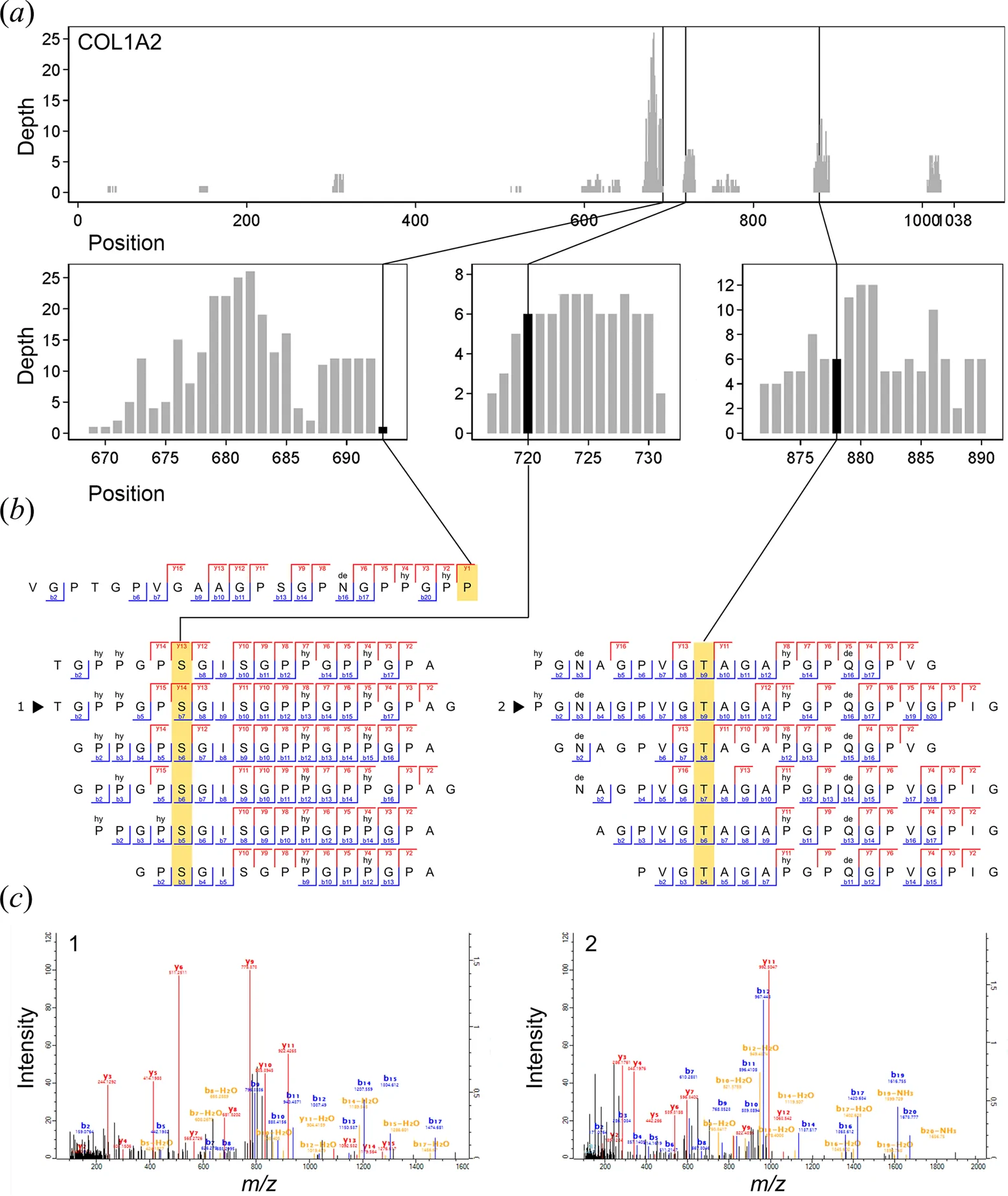
Details of the eland-specific set of substitutions in COL1A2 (693P, 720S and 878T) identified from the ochre-rich sediments at Klipdrift Shelter. (*a*) Peptide coverage depth along the mature region of the protein sequence, with details for the eland-specific set of substitutions (insets). (*b*) Supporting peptides, covering the eland-specific set of substitutions, were aligned, with the substitution position highlighted in yellow. hy, hydroxylated proline (P); de, deamidated amino acids (asparagine, N, and glutamine, Q). (*c*) MS2 spectra, confidently supporting the identification of peptides covering eland-specific substitutions. The respective patterns of positively identified fragment ions are marked with triangle symbols and the corresponding numbers in panel (*b*).

Based on these results, the observed peptide set can be parsimoniously attributed to *T. oryx*. The confident observation of positions 720S and 878T, sufficient to attribute the collagen to eland, both in PBE and PBD samples (each supported by ≥3 MS2 spectra; supplementary material, table S9), shows that the eland signal is consistently present across both layers. Although within the genus *Taurotragus*, *T. oryx* is the only species whose type I collagen sequence is currently available [63,75]; the current distribution of the other eland species, giant eland (*T. derbianus*), is limited to central Africa. Assuming the distribution of these two species in MSA Africa was the same as it is today, both the reconstructed sequence and known biogeography of African bovids lead to the conclusion that common eland is the sole most probable source for the COL1A2 found in KDS ochre-rich sediment samples.

### (b) Microscopic analysis

Only a small number of fragments of probable bone were identified in only a small number of sediment samples. Specifically, of the total of 15 samples analysed, only 4 contained any bone-like material, and these were represented by just one or two fragments (<1–3 mm, supplementary material, figures S13–S15). Most of these came from layer PBD (three samples) and none was identified from PBE. Overall, the low abundance and small size of these fragments indicate that they were rare in the samples analysed for proteomics. A summary of the list of samples and brief results of both optical and electron microscopic examinations are provided in supplementary material, table S12.

## 4. Discussion

Palaeoproteomic analysis identified type I collagen matching the common eland in two ochre-rich sediment samples from distinct Howiesons Poort layers (PBD and PBE). This finding aligns with previous macroscopic analyses of faunal remains at KDS, which also identified common eland [66], suggesting that this species was present at the site and may have held significance for its inhabitants. The observed near-complete deamidation of asparagine (N) and glutamine (Q) is consistent with long-term diagenetic alteration, supporting the ancient origin of the collagen proteins [80], even when taking into account that partial deamidation can result from accidental or intentional heating processes [81–83], as well as from acid treatment [84] (see supplementary material for calculation of deamidation). The preservation of eland collagen type I in the ochre-rich sediments can tentatively be explained by the relatively high concentration of iron oxide in the material we investigated. Iron has been suggested to stabilize proteins through processes such as iron‑mediated radical cross‑linking and tissue fixation [85–87]. The systematic characterization of the exact mechanisms underlying the survival of collagen in sediments dated at about 60 ka, although highly relevant, requires further, dedicated research that falls beyond the scope of this study.

Analysis of ochre particles within a thin section containing a small patch of the ochre surface from PBE shows a diverse assemblage of ochre types [1], grain sizes and morphologies (supplementary material, figure S3). Numerous highly processed, rounded and sub-rounded particles (approx. 1 µm) are present alongside larger sub-rounded fragments (approx. 2–5 mm) (supplementary material, figure S4). This diversity, combined with the substantial quantity of ochre required to produce the surface, reflects the complexity of this feature and further supports the possibility that its formation is due to human action.

We propose two possible scenarios for the association of eland-derived proteins and ochre surface: (1) eland-derived protein was used as a component in an ochre mixture that was then deposited as part of the ochre surface, or (2) an ochre-covered eland hide was deposited at the site. While the hide itself decomposed and is no longer visible, the ochre used to treat it may have remained *in situ* and formed the ochre surface. Other, as yet unidentified, ochre-related practices may also have played a role in the formation of this feature. Further interpretations of these scenarios are provided in supplementary material, ‘Scenarios for the anthropogenic origin of eland protein’. Additional studies on the micromorphological nature of such ochre deposits, combined with a database enabling the identification of the specific tissue involved, may shed light on the behaviours associated with the deposition of this feature.

## (a) Potential and limitations

Archaeological sites and materials are valuable, yet finite sources of information. As such, it is not always feasible to collect samples in strict adherence to established protocols designed for ancient biomolecular studies [88]. Utilizing legacy samples for the purpose of recovering ancient DNA has previously been carried out successfully [89]. Similarly, in this study, we were able to successfully extract and confidently identify ancient proteins from legacy samples, despite those samples not being collected with proteomic analysis in mind.

While it is likely that palaeoproteomic analysis could be equally successful when performed on legacy samples from other sites, the accurate reconstruction of the origin of the proteins detected also requires access to the pertinent microstratigraphic data, useful to better understand formation processes, post-depositional impacts and the integrity of layers and features. Accordingly, whenever possible, recording the spatial coordinates of samples and conducting detailed microstratigraphic observations through micromorphological block sampling and thin-section analysis can address these challenges.

## 5. Conclusions

The information provided by biomolecular traces preserved in the archaeological record could significantly contribute to a more refined interpretation of a site's broader context, especially in the absence of visible evidence. Given their higher resistance to degradation compared with DNA, proteins may therefore represent the only taxonomically informative biomolecular evidence retrievable when DNA is no longer present.

In this study, we identified proteinaceous traces from ochre-rich sediments dated to approximately 60 000 years ago, recovered from the ochre surface feature within the Howiesons Poort Middle Stone Age deposits at Klipdrift Shelter in South Africa. The set of identified peptides parsimoniously matched the common eland (*T. oryx*), a species native to southern Africa and deeply revered in the San mythology. Considering this finding alongside ethnographic and experimental evidence for ochre use, the presence of eland collagen is most probably due to human action, likely involving both utilitarian practices and early symbolic behaviours. On its own, the single example of eland proteins associated with ground ochre presented in this study is not sufficient to support the ritual significance of eland in the MSA. However, repetition of our work at other MSA sites will provide a more robust test for the hypothesis that the symbolic significance of eland has deep roots.

Advances in instrumentation and data processing techniques, combined with experimentation on new materials such as sediments, are rapidly increasing the potential range of applications of palaeoproteomics in archaeology. These advances are likely to yield a clearer understanding of the source of protein residues in soil sediments, and by extension, ochre-rich deposits. This could open up avenues for further research in order to better understand past human activities, particularly when the conventional materials for palaeoproteomics studies, such as bone and teeth, are unavailable or unable to unravel such information.

## Supplementary Material



## Data Availability

All mass spectrometry proteomics data as well as the FASTA files have been deposited in the ProteomeXchange Consortium (http://proteomecentral.proteomexchange.org) via the PRIDE partner repository [79] with the dataset identifier PXD069507. The data can be accessed at https://www.ebi.ac.uk/pride/archive/projects/PXD069507/. Supplementary material is available online [90].
