# Supplementary material for: Palaeoproteomic investigation of archaeological sediments associated with ochre use in the Middle Stone Age of southern Africa
Source: Proc Biol Sci. 2026 Oct 21;293(2081):20253300. doi: 10.1098/rspb.2025.3300 (PMC13682889; doi:10.1098/rspb.2025.3300)
Supplement: Supplementary file 1 [file rspb.2025.3300.zip › rspb.2025.3300/rspb20253300_si_001.docx]

**Electronic Supplementary Materials**

**Title:** Palaeoproteomic investigation of archaeological sediments associated with ochre use in the Middle Stone Age of southern Africa

**Authors:** Zahra Haghighi, Takumi Tsutaya, Elizabeth Catherine Velliky, Simon James Armitage, Christopher Miller, Meaghan Mackie, Francesco d'Errico, Gaudry Troche, Jesper Velgaard Olsen, Karen L. van Niekerk, Christopher Stuart Henshilwood, Enrico Cappellini

*Proceedings of the Royal Society B. (DOI: 10.1098/rspb.2025.3300)*

**Table of Contents**

[Ochre occurrences at Middle Stone Age deposits of KDS	2](#_Toc228996428)

[Palaeoproteomics	7](#_Toc228996429)

[*AB digestion method*	8](#_Toc228996430)

[*Digestion-free method*	9](#_Toc228996431)

[*Peptide purification by StageTips*	9](#_Toc228996432)

[*Nano‑liquid chromatography–tandem mass spectrometry (nLC‑MS/MS)*	10](#_Toc228996433)

[*Data analysis and protein identification*	11](#_Toc228996434)

[*Sequence reconstruction*	22](#_Toc228996435)

[*Calculation of deamidation*	27](#_Toc228996436)

[Microscopic analysis	28](#_Toc228996437)

[Scenarios for the anthropogenic origin of eland protein	34](#_Toc228996438)

[The significance of eland in the lives of hunter-gatherers	36](#_Toc228996439)

[References	37](#_Toc228996440)

## **Ochre occurrences at Middle Stone Age deposits of KDS**

Klipdrift Shelter (KDS) is located approximately 150 km east of Cape Town in the De Hoop Nature Reserve (34˚27.09630 S, 20˚43.45820 E), South Africa [[1]](https://paperpile.com/c/Vuv3M7/1E02c) and is part of Klipdrift Complex (Figure S1, S2), which also includes Klipdrift Cave (KDC) and Klipdrift Cave Lower (KDCL).

**Figure S1**- **View of Klipdrift Complex, South Africa, comprising Klipdrift Shelter (KDS), Klipdrift Cave (KDC) and Klipdrift Cave Lower (KDCL).** Image courtesy of Ole F. Unhammer.

The closest source of ochre to the KDS is a ferricrete outcrop 400 m to the east, with numerous ochre outcrops occurring within 5–20 km of the site in the Bokkeveld shale deposits [[1]](https://paperpile.com/c/Vuv3M7/1E02c). A recent analysis of a broader assemblage of ochre artefacts from KDS shows elemental compositions similar to those from nearby sites such as Pinnacle Point [[2]](https://paperpile.com/c/Vuv3M7/1QosB). Although this can only be confirmed through systematic provenance studies, if supported, these findings may suggest that the inhabitant populations of these sites employed similar collection strategies, implying that their primary ochre resources were local and sub‑local deposits [[2]](https://paperpile.com/c/Vuv3M7/1QosB).

**Figure S2. Detailed stratigraphy of Klipdrift shelter, showing the location of excavated quadrates and layers.** Units refer to the stratigraphic layers and are alphabetically ordered name codes. Layers from which proteomic samples were collected are indicated by the star symbols. Images courtesy of Magnus M. Haaland.

The bulk of the ochre assemblage comes from the densely occupied and well-preserved Howiesons Poort (HP) layers, PBA/PBB-PCA. Layer PCA contains the largest amount of ochre, as indicated by the number of pieces (n = 206), total weight (402.3 g), and ochre number per excavated volume (0,86700 pieces per litre) [[2]](https://paperpile.com/c/Vuv3M7/1QosB). In terms of artefact density per excavated volume, PCA has the highest density, followed by PBD, then PBA/PBB and lastly PBE (Table S1).

**Table S1-** KDS ochre counts (n) and weights by stratigraphic layer with excavated volume percentages (calculated in cm3).

| **Layer** | **Ochre (n)** | **% of total** | **Ochre (g)** | **% of total** | **Litres** | **cm^3^** | **n/cm^3^** | **g/cm^3^** | **n/ volume** | **g/ volume** |
| --- | --- | --- | --- | --- | --- | --- | --- | --- | --- | --- |
| **PAY** | 17 | 2.80% | 33 | 2.50% | 174.9 | 174 900 | 0.0001 | 0.00019 | 0.0972 | 0.18868 |
| **PAZ** | 16 | 2.70% | 33.7 | 2.60% | 132 | 132 000 | 0.00012 | 0.00026 | 0.12121 | 0.2553 |
| **PBA/**  **PBB** | 129 | 21.60% | 242.4 | 18.60% | 355.3 | 355 300 | 0.00036 | 0.00068 | 0.36307 | 0.68224 |
| **PBC** | 25 | 4.20% | 96.41 | 7.40% | 193.6 | 193 600 | 0.00013 | 0.0005 | 0.12913 | 0.49799 |
| **PBD** | 85 | 14.20% | 185.85 | 14.30% | 226.6 | 226 600 | 0.00038 | 0.00082 | 0.37511 | 0.82017 |
| **PBE** | 54 | 9.00% | 138.4 | 10.60% | 210.1 | 210 100 | 0.00026 | 0.00066 | 0.25702 | 0.65873 |
| **PCA** | 206 | 34.50% | 402.29 | 30.80% | 237.6 | 237 600 | 0.00087 | 0.00169 | 0.867 | 169,314 |
| **PCA/**  **PCB** | 9 | 1.50% | 47.72 | 3.70% | 108.9 | 108 900 | 0.00008 | 0.00044 | 0.08264 | 0.4382 |
| **PCB** | 8 | 1.30% | 4.76 | 0.40% | 17.6 | 17 600 | 0.00045 | 0.00027 | 0.45455 | 0.27045 |
| **PDA** |  | 0.00% |  | 0.00% | 16.5 | 16 500 | 0 | 0 | 0 | 0 |
| **PDB** | 1 | 0.20% | 4.6 | 0.40% | 11 | 1 1000 | 0.00009 | 0.00042 | 0.09091 | 0.41818 |

Layer PBE contains the highest number of artefacts bearing ochre residues (n = 58), one of which was the early/archaic hominin tooth that was ‘…covered with ochre, possibly from the powder and tiny pieces found throughout the base of the unit’ [[3]](https://paperpile.com/c/Vuv3M7/T931G). Additionally, numerous patches of ochre powder were recovered throughout the HP layers at KDS in different sizes, textures (rounded, sub-rounded and sub-angular) and conglomerations. In layer PBE, there is a laterally continuous ochre surface. Even though smaller patches of ochre are found throughout the HP layers, the size of the ochre layer in PBE is exceptional. In a thin-section containing a small patch of the ochre surface from PBE (Figure S3), numerous highly processed, rounded and sub-rounded ~1 µm ochre particles, as well as many larger (~2-5 mm) sub-rounded pieces of ochre are present within this thin-section and throughout the ochre surface (Figure S4). The substantial amount of ochre required to create such a surface, the presence of different grain morphologies, and the use of different ochre types [[1]](https://paperpile.com/c/Vuv3M7/1E02c) strongly supports the anthropogenic origin of this feature. In particular, macroscopic ochre analyses conducted at the site indicate that, of approximately 597 ochre artefacts, 29 exhibit anthropogenic traces consistent with pigment extraction [[2]](https://paperpile.com/c/Vuv3M7/1QosB).

**Figure S3- View of sample selected for thin sections of ochre surface from layer PBE.** Top: viewed in plane polarised light (PPL) and Bottom: crossed polarised light (XPL).

**Figure S4- Backscattered Electron (BSE) images of PBE thin section (Figure S3).** Top: view of numerous iron-rich particles highlighted in red forming a laterally continuous stringer. Bottom: close-up view showing highly processed, rounded and sub-rounded ochre particles <2 µm in size. Images were acquired using a secondary electron (SE) detector at 15 kV and 8.4 mm working distance.

## **Palaeoproteomics**

A summary of the samples used for proteomic analysis is provided in Table S2. The macroscopic and microscopic images of these samples are shown in Supplementary Figure S5.

**Table S2-** Overview of ochre-rich sediment samples collected for palaeoproteomics experiments, dating to between 60-64 ka [[1]](https://paperpile.com/c/Vuv3M7/1E02c). Samples were assigned two types of identifiers. The Sample ID includes the name of the layer and the quadrant from which the sample was collected. The LC-MS/MS Sample ID refers to the number assigned to each sample for mass spectrometry analysis.

| **Sample ID.** | **LC-MS/MS sample ID.** | **Layer** | **Quadrant** | **Excavation season** |
| --- | --- | --- | --- | --- |
| **PBD_S28b** | 2060, 2063, 2068, 2072, 2159, 2160 | PBD | S28b | 2011 |
| **PBE_S29b** | 2061, 2064, 2069, 2073 | PBE | S29b | 2011 |
| **PBD_S30a** | 2162 | PBD | S30a | 2012 |
| **PCA_R28d** | 2163 | PCA | R28d | 2012 |
| **PBA/PBB_R31a** | 2164 | PBA/PBB | R31a | 2013 |

**Figure S5- Macroscopic and microscopic images of samples analysed for proteomics.** a) PBD_S28b, b) PBD_S30a, c) PCA_R28d and d) PBA/PBB_R31a. Ochre conglomerates were collected from ochre-rich sediment samples; panels (b-d) show the conglomerates after extraction from the sediment, while panel (a) illustrates the appearance of samples prior to collection. As sample PBE_S29b was not photographed in the laboratory, a microscopic image from a comparable ochre conglomerate from the same stratigraphic layer and an adjacent quadrant (PBE_S28b) is instead provided.

### ***AB digestion method***

A new method, based on the bone protein extraction protocols developed by Sawafuji et al. [[4]](https://paperpile.com/c/Vuv3M7/cgI7X) and Tsutaya et al. [[5]](https://paperpile.com/c/Vuv3M7/nAa6p), was developed to extract proteins from sediment samples under different denaturing conditions. This involves sequential treatment of samples with acidic and basic solutions, hence the name ‘AB’, followed by treatment with chaotropic agents and digestion with trypsin. Two samples, PBD_S28b and PBE_S29b, were processed using AB digestion method. Details are provided in the main text and Figure S6.

### ***Digestion-free method***

This method follows the previously established protocol [[6,7]](https://paperpile.com/c/Vuv3M7/GGUg2+gmx3u). The ochre-rich sediment samples were immersed in 500 μL of 10% TFA overnight at 4 ℃. After the centrifuge (10 000 ×g, 5 min), the supernatant was collected and the pellet was immersed in 500 μL of 10% TFA overnight at 4 ℃ again. After the centrifuge (10 000 ×g, 5 min), the supernatant was collected and combined with the previous fraction. HCl was also used instead of TFA for the sample PBD (PBD_S28b_HCl). The dissolved solution of PBE (PBE_S29b_TFA) was filtered using a molecular weight cut off filter (Amicon 30K, Merck Millipore, USA). The resultant dissolved solutions were directly purified and desalted using C18 StageTips, consisting of stacked two layers of C18 membrane (CDS Analytical, USA). All samples from layers PBA/PBB, PBD, PBE, and PCA were processed using the digestion-free method. All batches were prepared alongside experimental blanks that did not contain any sample.

### ***Peptide purification by StageTips***

Dissolved or digested samples were purified and desalted using in-house-made StageTips with a C18 membrane [[8]](https://paperpile.com/c/Vuv3M7/4EKEe). The C18 membrane was washed with 150 μL methanol and equilibrated with 150 μL of 80% ACN containing 0.1% TFA and then 150 μL of 0.1% TFA. The sample solution was incrementally added and passed through the C18 membrane. After passing the sample, the C18 membrane was washed twice with 150 μL of 0.1% TFA.

**Figure S6- A step-by-step visualisation of AB digestion method.**

### ***Nano‑liquid chromatography–tandem mass spectrometry (nLC‑MS/MS)***

Samples were eluted from the StageTips into a 96 well MS plate using 30 μL 40% ACN, 0.1% formic acid (FA; Sigma-Aldrich, Germany) in LC–MS grade water (18.2 MΩ·cm resistivity). Samples were placed in a vacuum centrifuge at 40 °C until approximately 3 μL of solution was left and then rehydrated with 5 μL of 0.1% TFA, 5% ACN. Samples were then separated on a 15 cm column (75 μm inner diameter) in-house laser pulled and packed with 1.9 μm C18 beads (Dr. Maisch, Germany) on an EASY-nLC 1200 (Proxeon, Denmark) connected to an Exploris 480 Orbitrap mass spectrometer (Thermo Fisher Scientific, Germany) on a 77 min gradient. Buffer A was 0.1% FA in Milli-Q water (18.2 MΩ·cm resistivity), and the peptides were separated with increasing buffer B (80% ACN and 0.1% FA), going from 5% to 30% in 50 minutes, 30% to 45% in 10 minutes, 45% to 80% in 2 minutes, held at 80% for 5 minutes before dropping back down to 5% in 5 minutes and held for 5 minutes. The flow rate was 250 nL/min. The column temperature was kept at 40 °C. A wash-blank method using 0.1% TFA and 5% ACN solution was run between each sample to prevent cross-contamination.

The Exploris 480 was operated in data-dependent top-10 mode. The spray voltage was 2 kV, the S-lens RF level was 40%, and the ion transfer tube was heated at 275 °C. Full scan mass spectra were recorded at a resolution of 120 000 at *m/z* 200 over the *m/z* range 350–1400 with an injection time of 25 ms and an AGC target of 300%. Fragmentation was performed by HCD with normalised collision energy (NCE) set to 30 (on Thermo’s 0-100 scale and displayed as ‘30%’ in the instrument software). Scans were acquired at a resolution of 60 000 between *m/z* 100 and precursor *m/z* + 20. The isolation window was 1.2 *m/z* and an MS2 AGC was 200%. Ions were excluded if not above 2e4 intensity, with a charge state between 2 and 6, or detected as isotopologues. Fragmented ions were dynamically excluded for 20 seconds.

### ***Data analysis and protein identification***

The resulting raw files were analysed using MaxQuant software version 2.6.3.0. The parameter “Enzyme” was set to trypsin for digested samples. Two types of searches with “Specific” or “Unspecific” protease specificity were conducted, and undigested fractions were only included in the unspecific search. Oxidation (M), deamidation (NQ), pyro-Glu (EQ), and hydroxylation (P) were set as variable modifications. Carbamidomethylation (C) was set as a fixed modification for the digested fractions. Up to one missed cleavage was allowed per peptide for the digested samples. Error tolerances were kept at the default for Orbitrap mass spectrometers. Peptide and protein false discovery rates (FDR) were set to 1%. Proteins with at least 2 different non-overlapping peptides were regarded as confident identification, combined with manual examination of the MS/MS spectra. Contaminant proteins were assessed using the contamination.fasta provided by MaxQuant which includes common laboratory contaminants. These proteins were excluded from further analysis.

First, digested samples (PBD_S28b_S1, PBD_S28b_S2, PBD_S28b_P, PBE_S29b_S1, PBE_S29b_S2, and PBE_S29b_P) were searched against a Swiss-Prot database (downloaded 18 March 2022, ~568 000 protein entries) with specific trypsin cleavages, to tentatively identify the protein residues within the sediment samples without inflating the search space. This first search resulted in the identification of mammalian collagen alpha-1 (COL1A1) and -2 (COL1A2) type I proteins (Table S3).

**Table S3.** Identified proteins from the ochre-rich sediment samples in the first MaxQuant search with the Swiss-Prot database. Coverage and Score correspond to the result from the entire samples.

| **Leading Razor Protein** | **Species Name** | **Razor+Unique peptide** | | | | | | | | | | **Coverage (total)** | **Score**  **(total)** |
| --- | --- | --- | --- | --- | --- | --- | --- | --- | --- | --- | --- | --- | --- |
|  |  | **Total** | **Blank2_S1** | **Blank2_S2** | **Blank2_P** | **PBD_S1** | **PBD_S2** | **PBD_P** | **PBE_S1** | **PBE_S2** | **PBE_P** |  |  |
| Q9HWK6  Lysyl endopeptidase | *Pseudomonas aeruginosa* | 11 | 11 | 1 | 0 | 6 | 4 | 1 | 2 | 0 | 1 | 29.4 | 323.31 |
| P15636  Protease 1 | *Achromobacter lyticus* | 10 | 0 | 7 | 8 | 2 | 1 | 1 | 3 | 0 | 1 | 16.5 | 323.31 |
| P04264  Keratin, type II cytoskeletal 1 | *Homo sapiens* | 10 | 0 | 3 | 8 | 1 | 0 | 0 | 3 | 1 | 0 | 14.3 | 99.327 |
| P00761  Trypsin | *Sus scrofa* | 6 | 5 | 6 | 6 | 5 | 3 | 4 | 4 | 3 | 2 | 32.9 | 323.31 |
| C0HJN9  Collagen alpha-1(I) chain (Fragments) | *Equus sp.* | 5 | 0 | 0 | 0 | 0 | 0 | 0 | 4 | 1 | 2 | 9.6 | 257.97 |
| P02465  Collagen alpha-2(I) chain | *Bos taurus* | 5 | 0 | 0 | 0 | 0 | 0 | 0 | 5 | 0 | 0 | 5.3 | 48.786 |
| P35527  Keratin, type I cytoskeletal 9 | *Homo sapiens* | 3 | 0 | 0 | 1 | 2 | 0 | 0 | 2 | 0 | 0 | 5.9 | 133.22 |
| P16867  Histone H2B.3 | *Volvox carteri* | 2 | 2 | 2 | 2 | 2 | 1 | 1 | 1 | 0 | 2 | 12.1 | 43.161 |

Thus, a database containing 711 mammalian complete or partial COL1A1 and COL1A2 sequences registered in UniProt (downloaded 8 October 2024) was constructed. Additionally, mature COL1A1 and COL1A2 sequences from 44 bovid species naturally distributed in Africa, published by Le Meillour et al. (2023) [[9]](https://paperpile.com/c/Vuv3M7/JZWkX), which is the updated collagen database created by Janzen et al. (2021) [[10]](https://paperpile.com/c/Vuv3M7/o8PdG), were added to the dataset. All digested and undigested samples were searched against this database with unspecific protease cleavages for the final identification. Since the samples analysed here derive from ~60‑ka archaeological sediments, they are therefore expected to be highly degraded. As such, the second MaxQuant analysis was performed using an unspecific search. An unspecific search is better suited for recovering a broader range of ancient proteins, including those generated through random, non‑enzymatic fragmentation under burial conditions. To confirm the species identification, all identified peptides underwent BLASTp searches through the NCBI database (BLAST: Basic Local Alignment Search Tool) [[11]](https://paperpile.com/c/Vuv3M7/sAMkv) to validate species identifications and to rule out conserved peptides between species (Table S4-S8). This second search resulted in the identification of COL1A1 and COL1A2, and the results were used for further analyses.

**Figure S7- Histogram showing the peptide length distribution of proteins identified from ochre-rich sediment samples.** These peptides were identified from undigested fractions.

**Table S4-** Details of the samples analysed via proteomics and the respective extraction methods. Razor and unique peptides and covered AA (checked) are obtained for the common eland collagen proteins (COL1A1 and COL1A2) and represent the number of identified razor+unique (R+U) peptides and identified amino acid residues validated by MS2 spectra respectively. Samples were assigned two types of identifiers. The Sample ID includes the name of the layer, the quadrant from which the sample was collected, and the fraction or extraction agent used. The LC_MS/MS Sample ID refers to the number assigned to each sample for mass spectrometry analysis.

| **Experiment No.** | **Sample ID.** | **Archaeological sample** | **Quantity (mg)** | **Extraction method** | **Fraction** | **COL1A1**  **R+U peptides** | **COL1A2**  **R+U peptides** | **COL1A1**  **Covered AA (checked)** | **COL1A2**  **Covered AA (checked)** | **LC-MS/MS sample ID.** |
| --- | --- | --- | --- | --- | --- | --- | --- | --- | --- | --- |
| 1 | Blank1_TFA | _ | _ | Digestion-free / TFA | _ | _ | _ | _ | _ | 2059 |
| 1 | PBD_S28b_TFA1 | PBD_S28b | 96.5 | Digestion-free / TFA | _ | 165 | 85 | 123 | 48 | 2060 |
| 1 | PBE_S29b_TFA | PBE_S29b | 155.0 | Digestion-free / TFA | _ | 92 | 17 | 29 | 6 | 2061 |
| 2 | Blank2_S1 | _ | _ | AB digestion | S1 | _ | _ | _ | _ | 2062 |
| 2 | PBD_S28b_S1 | PBD_S28b | 127.6 | AB digestion | S1 | 0 | 0 | 0 | 0 | 2063 |
| 2 | PBE_S29b_S1 | PBE_S29b | 132.7 | AB digestion | S1 | 206 | 76 | 59 | 13 | 2064 |
| 2 | Blank2_S2 | _ | _ | AB digestion | S2 | _ | _ | _ | _ | 2067 |
| 2 | PBD_S28b_S2 | PBD_S28b | _ | AB digestion | S2 | 0 | 0 | 0 | 0 | 2068 |
| 2 | PBE_S29b_S2 | PBE_S29b | _ | AB digestion | S2 | 0 | 0 | 0 | 0 | 2069 |
| 2 | Blank2_P | _ | _ | AB digestion | P | _ | _ | _ | _ | 2071 |
| 2 | PBD_S28b_P | PBD_S28b | _ | AB digestion | P | 0 | 0 | 0 | 0 | 2072 |

**Table S4-** *Continued.*

| **Experiment No.** | **Sample ID.** | **Archaeological sample** | **Quantity (mg)** | **Extraction method** | **Fraction** | **COL1A1**  **R+U peptides** | **COL1A2**  **R+U peptides** | **COL1A1**  **Covered AA (checked)** | **COL1A2**  **Covered AA (checked)** | **LC/MS ID.** |
| --- | --- | --- | --- | --- | --- | --- | --- | --- | --- | --- |
| 2 | PBE_S29b_P | PBE_S29b | _ | AB digestion | P | 166 | 94 | 57 | 24 | 2073 |
| 3 | Blank3_TFA | _ | _ | Digestion-free / TFA | _ | _ | _ | _ | _ | 2158 |
| 3 | PBD_S28b_TFA2 | PBD_S28b | 127.0 | Digestion-free / TFA | _ | 15 | 10 | 4 | 3 | 2159 |
| 3 | PBD_S28b_TFA2_test | PBD_S28b | _ | Digestion-free / TFA | _ | 57 | 0 | 20 | 9 | 2159 |
| 3 | PBD_S28b_HCl | PBD_S28b | 118.0 | Digestion-free / HCL | _ | 10 | 0 | 2 | 0 | 2160 |
| 4 | Blank4_TFA | _ | _ | Digestion-free / TFA | _ | _ | _ | _ | _ | 2161 |
| 4 | PBD_S30a_TFA | PBD_S30a | 70.0 | Digestion-free / TFA | _ | 0 | 0 | 0 | 0 | 2162 |
| 4 | PCA_R28d_TFA | PCA_R28d | 52.0 | Digestion-free / TFA | _ | 0 | 0 | 0 | 0 | 2163 |
| 4 | PBA/PBB_R31a_TFA | PBA/PBB_R31a | 32.0 | Digestion-free / TFA | _ | 0 | 0 | 0 | 0 | 2164 |

**Table S5.** Summary statistics of the identified proteins from ochre-rich sediment samples. *R+U peptides:* The number of identified razor+unique peptides. *AA:* amino acid*, Coverage:* number and coverage of identified amino acid residues with spectral support. *Length:* Length of the protein. *Depth:* depth of the covered AA residues. *Note:* The MaxQuant output for the COL1A1 entry lists *Tragelaphus strepsiceros* as the leading razor protein. However, the identified sequence for COL1A1 is identical across *T. oryx* and all four species within the genus Tragelaphus. Therefore, it is appropriate to consider the organisms represented by COL1A1 as multiple species, including *T. oryx*.

| **Leading Razor Protein** | **Species Name** | **R+U peptides** | **AA** | **Coverage** | **Length** | **Depth** | | | **Note** |
| --- | --- | --- | --- | --- | --- | --- | --- | --- | --- |
|  |  |  |  |  |  | **Max** | **Average** | **Entire Mean** |  |
| COL1A1_Bovinae_  Tragelaphus_strepsiceros* | *Tragelaphus strepsiceros*  (Greater kudu) | 185 | 306 | 28.9 | 1057 | 35 | 6.343 | 1.836 | *Neotragus moschatus*, *Neotragus pygmaeus*, *Philantomba maxwellii*, *Taurotragus oryx*, *Tragelaphus buxtoni*, *Tragelaphus imberbis*, *Tragelaphus strepsiceros* have the same sequence in the identified positions. |
| COL1A2_Bovinae_  Taurotragus_oryx* | *Taurotragus oryx* (Common eland) | 69 | 171 | 16.5 | 1038 | 26 | 4.058 | 0.669 | Unique to common eland |
| A0A287CWH6  Collagen alpha-1(I) chain | *Ictidomys tridecemlineatus* (Thirteen-lined ground squirrel) | 7 | 28 | 2.2 | 1268 | 6 | 3.286 | 0.073 | Wrong protein sequence annotation in UniProt. |
| C0HJP8  Collagen alpha-2(I) chain | *Toxodon sp.* (Toxodon) | 4 | 16 | 1.8 | 908 | 4 | 2.313 | 0.041 | No diagnostic position covered |
| A0A1S2ZPI9  Collagen alpha-2(I) chain | *Erinaceus europaeus* (Western European hedgehog) | 3 | 25 | 1.8 | 1368 | 2 | 1.520 | 0.028 | No diagnostic position covered |
| A0A4X1USL6  Collagen alpha-1(I) chain | *Sus scrofa*  (Domestic pig) | 3 | 14 | 1.0 | 1449 | 3 | 2.000 | 0.019 | Only one diagnostic peptide |
| A0A835ZZ30  Collagen alpha-1(I) chain | *Ovis aries*  (Domestic sheep) | 3 | 11 | 0.7 | 1520 | 3 | 2.545 | 0.018 | Propeptide |

**Table S5.** *Continued.*

| **Leading Razor Protein** | **Species Name** | **R+U peptides** | **AA** | **Coverage** | **Length** | **Depth** | | | **Note** |
| --- | --- | --- | --- | --- | --- | --- | --- | --- | --- |
|  |  |  |  |  |  | **Max** | **Average** | **Entire Mean** |  |
| A0A8C0ZM04  Collagen alpha-1(I) chain | *Castor canadensis* (American beaver) | 3 | 12 | 0.8 | 1453 | 3 | 2.667 | 0.022 | No diagnostic position covered |
| P02466  Collagen alpha-2(I) chain | *Rattus norvegicus* (Rat) | 3 | 19 | 1.4 | 1372 | 3 | 1.632 | 0.023 | Propeptide |
| A0A6J0Z691  Collagen alpha-1(I) chain | *Odocoileus virginianus texanus* (Texas white-tailed deer) | 2 | 10 | 0.7 | 1463 | 2 | 1.400 | 0.010 | No diagnostic position covered |
| A0AAJ7GGU8  Collagen alpha-1(I) chain | *Rhinopithecus bieti* (Black snub-nosed monkey) | 2 | 9 | 0.6 | 1464 | 2 | 1.333 | 0.008 | Only one diagnostic peptide |
| S7N9H2  Collagen alpha-2(I) chain | *Myotis brandtii* (Brandt's bat) | 2 | 5 | 0.4 | 1328 | 2 | 2.000 | 0.008 | No diagnostic position covered |

* These two proteins were identified using the database published by Le Meillour et al. (2023) [[9]](https://paperpile.com/c/Vuv3M7/JZWkX), which includes COL1A1 and COL1A2 sequences of 44 bovid species. The remaining proteins were identified using a dataset containing 711 mammalian COL1A1 and COL1A2 sequences registered in UniProt (downloaded 8 October 2024).

**Table S6-** Summary of the identified proteins and the respective number of identified razor+unique peptides from each of the ochre-rich sediment samples, their coverage and score in the second MaxQuant search with the restricted mammalian collagen database.

| **Leading Razor Protein**  **/** **Species Name** | **Razor+Unique Peptides** | | | | | | | | | | | | | | | **Coverage** | **Score** |
| --- | --- | --- | --- | --- | --- | --- | --- | --- | --- | --- | --- | --- | --- | --- | --- | --- | --- |
|  | Total | PBA/PBB | PCA | PBD | PBD | PBD | PBD | PBD | PBD | PBD | PBD | PBE | PBE | PBE | PBE |  |  |
|  |  | R31a  TFA | R28d  TFA | S28b  P | S28b  S1 | S28b  S2 | S28b  HCl | S28b  TFA2_test | S28b  TFA1 | S28b  TFA2 | 30a  TFA | S29b  P | S29b  S1 | S29b  S2 | S29b  TFA |  |  |
| COL1A1_Bovinae_  Tragelaphus_  strepsiceros* | 212 | 0 | 0 | 0 | 0 | 0 | 2 | 20 | 123 | 4 | 0 | 57 | 59 | 0 | 29 | 47 | 323.31 |
| COL1A2_Bovinae_  Taurotragus_oryx* | 80 | 0 | 0 | 0 | 0 | 0 | 0 | 9 | 48 | 3 | 0 | 24 | 13 | 0 | 6 | 30.5 | 323.31 |
| COL1A1_A0A287CWH6  *Ictidomys*  *tridecemlineatus* | 7 | 0 | 0 | 0 | 0 | 0 | 0 | 0 | 7 | 0 | 0 | 1 | 0 | 0 | 0 | 3 | 94.198 |
| COL1A1_A0A835ZZ30  *Ovis aries* | 7 | 0 | 0 | 0 | 0 | 0 | 0 | 3 | 1 | 1 | 1 | 2 | 0 | 0 | 0 | 5.5 | 71.303 |
| COL1A2_C0HJP8  *Toxodon sp* | 5 | 0 | 0 | 0 | 0 | 0 | 0 | 0 | 5 | 0 | 0 | 0 | 0 | 0 | 0 | 2.6 | 45.324 |
| COL1A2_A0A1S2ZPI9  *Erinaceus europaeus* | 4 | 0 | 0 | 0 | 0 | 0 | 0 | 0 | 1 | 0 | 0 | 4 | 0 | 0 | 0 | 2.6 | 58.958 |
| COL1A1_  A0A8C0ZM04  *Castor canadensis* | 4 | 0 | 0 | 0 | 0 | 0 | 0 | 0 | 0 | 0 | 0 | 4 | 2 | 0 | 0 | 2.8 | 29.805 |
| COL1A2_A0AA41TAK5  *Sciurus carolinensis* | 4 | 0 | 0 | 0 | 0 | 1 | 1 | 1 | 0 | 0 | 0 | 0 | 1 | 0 | 0 | 3.1 | 21.493 |
| COL1A2_C0HJN8  *Tapirus terrestris* | 4 | 0 | 0 | 0 | 0 | 0 | 0 | 4 | 0 | 0 | 0 | 0 | 0 | 0 | 0 | 5.2 | 21.511 |

**Table S6-** *Continued.*

| COL1A1_A0A4X1USL6  *Sus scrofa* | 3 | 0 | 0 | 0 | 0 | 0 | 0 | 0 | 2 | 0 | 0 | 0 | 1 | 0 | 0 | 1.4 | 29.378 |
| --- | --- | --- | --- | --- | --- | --- | --- | --- | --- | --- | --- | --- | --- | --- | --- | --- | --- |
| COL1A1_A0A8C4PSR9  *Equus asinus* | 3 | 0 | 0 | 0 | 0 | 0 | 0 | 2 | 0 | 0 | 0 | 1 | 0 | 0 | 0 | 2.9 | 19.321 |
| COL1A2_P02466  *Rattus norvegicus* | 3 | 0 | 0 | 0 | 0 | 0 | 0 | 0 | 3 | 0 | 0 | 0 | 1 | 0 | 0 | 1.5 | 28.019 |
| COL1A1_A0A6J0Z691  *Odocoileus virginianus texanus* | 2 | 0 | 0 | 0 | 0 | 0 | 0 | 0 | 0 | 0 | 0 | 1 | 1 | 0 | 0 | 1.6 | 19.598 |
| COL1A2_A0A8B7PYF0  *Hipposideros armiger* | 2 | 0 | 0 | 0 | 0 | 0 | 0 | 0 | 1 | 0 | 0 | 1 | 0 | 0 | 0 | 2.9 | 13.075 |
| COL1A1_Q28396  *Equus caballus* | 2 | 0 | 0 | 0 | 0 | 0 | 0 | 1 | 0 | 0 | 0 | 0 | 0 | 0 | 1 | 1.8 | -2 |
| COL1A1_A0AAJ7GGU8  *Rhinopithecus bieti* | 2 | 0 | 0 | 0 | 0 | 0 | 0 | 0 | 0 | 0 | 0 | 1 | 1 | 0 | 0 | 1.3 | -2 |
| COL1A1_F7ESN3  *Ornithorhynchus anatinus* | 2 | 0 | 0 | 0 | 0 | 0 | 0 | 0 | 1 | 0 | 0 | 1 | 0 | 0 | 0 | 1.7 | -2 |
| COL1A2_M3XR96  *Mustela putorius furo* | 2 | 0 | 0 | 0 | 0 | 0 | 0 | 2 | 0 | 0 | 0 | 0 | 0 | 0 | 0 | 2.1 | 14.58 |
| COL1A2_S7N9H2  *Myotis brandtii* | 2 | 0 | 0 | 0 | 0 | 0 | 0 | 0 | 0 | 0 | 0 | 2 | 0 | 0 | 0 | 1.6 | 14.701 |

*These two proteins were identified using the database published by Le Meillour et al. (2023) [[9]](https://paperpile.com/c/Vuv3M7/JZWkX), which includes 44 bovid species. The remaining proteins were identified using a dataset containing 711 mammalian COL1A1 and COL1A2 sequences registered in UniProt (downloaded 8 October 2024).

**Table S7.** Identified proteins from the experimental blank samples in the second MaxQuant search with the restricted mammalian collagen database.

| **Leading Razor Protein** | **Species Name** | **Razor+Unique Peptides** | | | | | | | **Coverage** | **Score** |
| --- | --- | --- | --- | --- | --- | --- | --- | --- | --- | --- |
|  |  | **Total** | **Blank1_TFA** | **Blank2_S1** | **Blank2_S2** | **Blank2_P** | **Blank3_TFA** | **Blank4_TFA** |  |  |
| P02453  Collagen alpha-1(I) chain | *Bos taurus* | 26 | 0 | 0 | 0 | 0 | 26 | 0 | 21.3 | 323.31 |
| A0A6P3HFZ4  Collagen alpha-2(I) chain | *Bison bison bison* | 16 | 0 | 0 | 0 | 0 | 16 | 0 | 16.1 | 323.31 |
| C0HJP0  Collagen alpha-2(I) chain  (Fragment) | *Equus sp.* | 7 | 0 | 0 | 0 | 0 | 7 | 0 | 12.9 | 227.89 |
| A0A2U3VH59  Collagen alpha-2(I) chain | *Odobenus rosmarus divergens* | 2 | 0 | 0 | 0 | 1 | 1 | 0 | 2.2 | 20.878 |
| A0AA41TAK5  Collagen alpha-2(I) chain | *Sciurus carolinensis* | 2 | 0 | 1 | 1 | 1 | 1 | 0 | 1.8 | 26.577 |
| C0HJN8  Collagen alpha-2(I) chain  (Fragment) | *Tapirus terrestris* | 2 | 0 | 0 | 0 | 0 | 2 | 0 | 4 | 119.61 |

**Table S8.** Summary of the total count of amino acids (AA) recovered from the ochre-rich sediment samples compared to the complete collagen proteins (COL1A1 and COL1A2) sequence of *Taurotragus oryx* published by Le Meillour et al. (2023) [[9]](https://paperpile.com/c/Vuv3M7/JZWkX).

| **Amino Acid** | **AA count in COL1A1 sequence** | | **AA count in COL1A2 sequence** | | **Molecular Weight (Da)** | **Explanation** |
| --- | --- | --- | --- | --- | --- | --- |
|  | **Entire region** | **Retrieved region** | **Entire region** | **Retrieved region** |  |  |
| Isoleucine (I)* | 9 | 3 | 19 | 2 | 131 | The R group includes a nonpolar, aliphatic side chain. |
| Valine (V)* | 18 | 5 | 36 | 8 | 117 | The R group includes a nonpolar, aliphatic side chain. |
| Leucine (L)* | 22 | 0 | 33 | 0 | 131 | The R group includes a nonpolar, aliphatic side chain. |
| Phenylalanine (F)* | 13 | 2 | 16 | 1 | 165 | The R group includes a nonpolar, aromatic side chain. |
| Tryptophan (W) | 0 | 0 | 0 | 0 | 204 | has both hydrophobic and hydrophilic properties due to its indole ring. |
| Alanine (A)* | 123 | 41 | 101 | 19 | 89 | The R group includes a small, nonpolar side chain. |
| Methionine (M)* | 7 | 1 | 4 | 0 | 149 | The R group includes a nonpolar, sulphur-containing side chain that repels water. |
| Proline (P)* | 241 | 103 | 210 | 53 | 115 | The R group includes a nonpolar, cyclic side chain. |
| Tyrosine (Y) | 5 | 0 | 3 | 0 | 181 | Has a polar hydroxyl group and a nonpolar aromatic ring. |
| Glycine (G) | 346 | 96 | 349 | 55 | 75 | The smallest amino acid can fit into hydrophobic or hydrophilic environments. |
| Serine (S) | 35 | 5 | 35 | 7 | 105 | The R group includes a polar hydroxyl group |
| Threonine (T) | 18 | 9 | 20 | 6 | 119 | The R group includes a polar hydroxyl group |
| Asparagine (N) | 10 | 3 | 25 | 5 | 132 | The R group includes a polar amide group |
| Glutamine (Q) | 30 | 9 | 24 | 3 | 146 | The R group includes a polar amide group |
| Glutamic Acid (E) | 49 | 11 | 44 | 7 | 147 | The R group includes a negatively charged carboxyl group. |
| Aspartic Acid (D) | 34 | 5 | 25 | 1 | 133 | The R group includes a negatively charged carboxyl group. |
| Histidine (H) | 3 | 0 | 8 | 0 | 155 | The R group includes a positively charged side chain. |
| Arginine (R) | 53 | 6 | 55 | 3 | 174 | The R group includes a positively charged guanidinium group. |
| Lysine (K) | 39 | 7 | 31 | 1 | 146 | The R group includes a positively charged amino group. |
| Total AA residues | 1055 | 306 | 1038 | 171 |  | |

* Although there are numerous scales to classify amino acid residues based on their interaction with their surrounding environment (e.g. aqueous environment), these amino acids are generally considered to have hydrophobic properties [[12,13]](https://paperpile.com/c/Vuv3M7/jGp0N+3cgF6).

### ***Sequence reconstruction***

Ancient protein sequences were reconstructed from the outputs of the final MaxQuant unspecific search using the mammalian collagen type I and African bovid COL1A1 and COL1A2 database. Results from different fractions were combined to create a unified consensus sequence. For the five key sites supporting the eland assignment, peptides were identified at four of the positions in PBD and at all positions in PBE at the MS1 level (Figure S8). Most of these variants were also confirmed in both PBD and PBE through manual validation of the corresponding MS2 spectra (Table S9). In particular, these two positions are exclusively observed in eland (COL1A2: 720S and 878T) and both are confidently recovered from both samples at the MS2 level. Taken together, these results indicate that the eland signal is consistently present across both layers.

First, tandem mass spectra of all peptides were manually validated, and amino acid residues not supported by any peak were converted into “X” (unknown amino acid) in the sequence of the peptide. Peptides with a peptide score of <80 and identified from experimental blanks were not used for the sequence reconstruction. Second, validated sequences of all peptides of eland COL1A1 or COL1A2 were mapped onto the entire length of the subject proteins to create a table showing the position, corresponding amino acid, and depth indicating how many peptides cover the site (Figure 2, S9, S10, S11). A unified consensus sequence for eland COL1A1 and COL1A2 from the table was generated. In this sequence, positions not covered by any peptides are marked by "-", while positions where peptides were identified at the MS1 level but could not be confidently assigned from the MS2 fragmentation pattern are indicated by "X" (Data data_COL1A1 and data_COL1A2). These sequences were compared with COL1A1 or COL1A2 sequences of other African bovid species [[10]](https://paperpile.com/c/Vuv3M7/o8PdG). The R script reported by Xia et al., (2024) [[14]](https://paperpile.com/c/Vuv3M7/0uXk2) was modified and used for sequence reconstruction. The modified version of R script, together with the relevant data files is accessible via doi: 10.5281/zenodo.17549431.

Manual evaluation of peptide sequence assignments from MS2 spectra was performed according to the following deliberately stringent rules, designed to exclude any ambiguities. In other words, we only retained amino acids whose position in the sequence was unambiguously supported by the fragmentation patterns. For the same reason, we do not apply the collagen-specific rule that assumes every third amino acid is Glycine. For reference, the complete list of peptide sequences, including the manually verified amino-acid residues, has been uploaded as *Peptides_checked_sample.txt* in the link above.

As an illustrative example, consider the peptide GPPGPPGPVGK, which consists of 11 amino acid residues. For simplicity, we ignore b-ions here and assume that y-ion fragments are observed at the positions indicated by “|”: G|PP|G|P|P|G|PV|GK. Applying the rules used in this study and replacing residues that are not confidently sequenced by the MS2 data with “X” yields the following sequence: GXXGPPGXXXX.

First, the C-terminal blocks PV and GK were replaced by XX. This is because, based solely on the y-ion series shown above, we cannot exclude the alternative orders VP and KG, respectively. In tryptic digests, the C-terminus of identified peptides is expected to be K or R, but especially in ancient samples, proteolysis-independent taphonomic hydrolysis and other accidental cleavage events cannot be ruled out. For this reason, we chose a conservative approach and treated the C-terminal GK as ambiguous, and thus replaced both residues with X.

In contrast, the central GPPG segment is supported residue-by-residue by the observed fragmentation pattern, and we therefore considered this part of the sequence to be confidently determined.

For the N-terminal PP block, both residues are identical, and therefore no positional ambiguity arises from their order. Considering the 20 canonical amino acids, and ignoring the special case of Leu/Ile isomerism, there is no pair of identical residues for which the sum of their monoisotopic residue masses is exactly equal to the sum of the monoisotopic residue masses of a different pair of amino acids. Under typical high-resolution mass spectrometric conditions, a block of two identical residues that is bracketed by fragment ions on both sides (as in |PP| in the example above) can thus be regarded as determined in terms of sequence. However, in the context of ancient proteins, unusual post-translational modifications may be present, and at the level of single residues, we cannot guarantee a confident assignment. Also, local mass errors may occur. We therefore adopted a conservative rule that any stretch of two identical residues that is not individually supported at the single–amino-acid level by the fragmentation pattern is treated as ambiguous, and both residues are replaced by X.

Finally, for the N-terminal G, the identity of this residue can be inferred from the precursor (MS1) mass. Specifically, subtracting the monoisotopic mass of the y-ion corresponding to PPGPPGPVGK from the monoisotopic mass of the intact peptide uniquely yields the mass of a glycine residue. On this basis, we treated the N-terminal G as confidently assigned.

Compared to COL1A2, COL1A1 appears to be more conserved, with fewer amino acid substitutions across species. Analysis of reconstructed sequences of COL1A1 alone shows two positions with variations in amino acid: Threonine (T), Alanine (A), or Serine (S) in position 622, and Valine (V) or A in position 739. Specifically, the combination of T in position 622 and V in position 739 is found in the sequence of *Taurotragus oryx* (Figure S9, S10). Beyond *T. oryx*, this peptide combination is observed in one species of Antilopinae (*Neotragus moschatus*), and six species of Bovinae (*Bucyrus eurycerus*, *Tragelaphus buxtoni*, *T. imberbis*, *T. scriptus*, *T. spekii*, and *T. strepsiceros*). Of these, only four species are currently native to southern Africa: *N. moschatus*, *T. scriptus*, *T. spekii*, and *T. strepsiceros*.

**Figure S8- The depth of supporting peptides along the mature region of the a) COL1A1 and b) COL1A2 protein sequences.** Peptides are shown redundantly when obtained from samples originating from different layers (i.e., PBD and PBE), even if they share the same sequence. Furthermore, these results are based on the less stringent results without manual validation of PSMs. Consequently, the depth at the corresponding sites is greater than that shown in Figure 2 or Figure S10.

**Table S9- The number of peptides for which manual validation of the MS2 spectrum confirmed the presence of variants observed in eland collagen sequences (COL1A1 and COL1A2).** The results are presented separately for samples obtained from the layers PBD and PBE. Even if the identification included different PSMs, they were counted as a single entry if their sequences were identical.

| **Protein** | **COL1A1** | | **COL1A2** | | |
| --- | --- | --- | --- | --- | --- |
| **Amino acid Variant** | **622T** | **739V** | **693P** | **720S** | **878T** |
| PBD | 0 | 2 | 1 | 6 | 5 |
| PBE | 7 | 4 | 0 | 3 | 4 |
| Total | 7 | 6 | 1 | 6 | 6 |

**Figure S9- Sequence alignment of COL1A1 of naturally distributed African bovids published by Le Meillour et al. (2023)** [[9]](https://paperpile.com/c/Vuv3M7/JZWkX) **and ochre-rich sediment sample from KDS.** The 622T and 739V are the variants shared with but not specific to the common eland. Positions not covered by any peptide are indicated with “–”. Positions where peptides were detected at the MS1 level but could not be confidently assigned based on MS2 manual inspection are indicated with “X”. Positions confidently determined from MS2 spectra are represented by their corresponding amino‑acid abbreviations. Multiple sequence alignments were visualised using Jalview [[15]](https://paperpile.com/c/Vuv3M7/zwIl0).

**Figure S10-** **Details of substitutions (not specific to eland) in COL1A1 identified from the ochre-rich sediments.** Peptide coverage depth along the mature region of protein sequence is shown. Supporting peptides, covering these positions were aligned, with the substitution position highlighted in yellow. The respective patterns of positively identified fragment ions are marked with triangle symbols and the corresponding numbers.

**Figure S11- Sequence alignment of COL1A2 proteins of naturally distributed African bovids published by Le Meillour et al. (2023)** [[9]](https://paperpile.com/c/Vuv3M7/JZWkX) **and ochre-rich sediment sample from KDS.** The combination of 693P, 720S and 878T is unique to common eland. Positions not covered by any peptide are indicated with “–”. Positions where peptides were detected at the MS1 level but could not be confidently assigned based on MS2 manual inspection are indicated with “X”. Positions confidently determined from MS2 spectra are represented by their corresponding amino‑acid abbreviations. Multiple sequence alignments were visualised using Jalview [[15]](https://paperpile.com/c/Vuv3M7/zwIl0)

### ***Calculation of deamidation***

Deamidation is a spontaneous chemical modification and can be characterised by a mass shift of +0.984 Da. Deamidation occurs when asparagine (Asn or N) and glutamine (Gln or Q) residues convert to aspartic acid (Asp or D) and glutamic acid (Glu or E) respectively by losing an amino group [[16]](https://paperpile.com/c/Vuv3M7/vOTkV). As this process continues postmortem, the extent of deamidation can be used to assess protein degradation, and therefore authentication of endogenous proteins [[17]](https://paperpile.com/c/Vuv3M7/ZM4OT) or sample preservation [[18]](https://paperpile.com/c/Vuv3M7/OqJCN). However, as the deamidation rate can be affected by several factors other than time [[19]](https://paperpile.com/c/Vuv3M7/XqkH3), it cannot be used as a dating tool [[20]](https://paperpile.com/c/Vuv3M7/aBnb9). Although deamidation can occur during acid treatment, its impact is considered relatively minor compared with diagenetic effects [[21]](https://paperpile.com/c/Vuv3M7/Osal8). Given that Gln deamidation occurs at a slower rate compared to Asn deamidation, Gln deamidation extent can be used to assess ageing and protein authentication.

MaxQuant output evidence file was used for the calculation of the deamidation of N and Q. An R script, rDeamidation [[22]](https://paperpile.com/c/Vuv3M7/eV4zl), based on a Python script reported by Mackie et al. (2018) [[23]](https://paperpile.com/c/Vuv3M7/Qgm8t) was used. Deamidation rates were calculated for PSMs of both eland COL1A1/COL1A2 (Table S10) and proteins identified from the blanks (i.e., mostly potential contaminants, Table S11). Taken together, these two tables support an ancient origin for the eland proteins identified in the archaeological samples, and indicate that the sample preparation (e.g. acid treatment) had only a minimal impact on their observed deamidation extent.

**Table S10.** Deamidation rates of the common eland COL1A1 and COL1A2 calculated using an R script, rDeamidation [[22]](https://paperpile.com/c/Vuv3M7/eV4zl), based on a Python script by Mackie et al. [[23]](https://paperpile.com/c/Vuv3M7/Qgm8t). Due to the low Razor and unique identified peptides, PBD_S28b_HCl was omitted from deamidation calculation. (N: Asparagine, Q: Glutamine, SD: Standard Deviation, CI: Confidence Interval, and n: the number of peptides available for calculation of deamidation).

| **Sample ID.** | **N** | | | | | **Q** | | | | |
| --- | --- | --- | --- | --- | --- | --- | --- | --- | --- | --- |
|  | **Mean** | **SD** | **95% CI, lower** | **95% CI, upper** | **n** | **Mean** | **SD** | **95% CI, lower** | **95% CI, upper** | **n** |
| PBD_S28b_TFA1 | **100.0** | **0.0** | **100** | **100** | **81** | **87.3** | **8.6** | **66.7** | **100** | **17** |
| PBD_S28b_TFA2 | 100.0 | 0.0 | 100 | 100 | 1 | 0.0 | 0.0 | NA | NA | 1 |
| PBD_S28b_TFA2_test | 27.4 | 18.2 | 0 | 59.7 | 7 | **1.6** | **1.2** | **0** | **4.0** | **12** |
| PBE_S29b_TFA | 100.0 | 0.0 | 100 | 100 | 7 | 100.0 | 0.0 | – | – | 2 |
| PBE_S29b_S1 | **93.6** | **6.3** | **77.8** | **100** | **17** | **100.0** | **0.0** | **100** | **100** | **17** |
| PBE_S29b_P | **100.0** | **0.0** | **100** | **100** | **17** | **100.0** | **0.0** | **100** | **100** | **12** |

**Table S11.** Deamidation rates of contaminant proteins recovered from the blanks, calculated

using an R script, rDeamidation [[22]](https://paperpile.com/c/Vuv3M7/eV4zl), based on a Python script by Mackie et al. [[23]](https://paperpile.com/c/Vuv3M7/Qgm8t). (N: Asparagine, Q: Glutamine, SD: Standard Deviation, CI: Confidence Interval, and n: the number of peptides available for calculation of deamidation).

| **Blank ID.** | **N** | | | | | | **Q** | | | | |
| --- | --- | --- | --- | --- | --- | --- | --- | --- | --- | --- | --- |
|  | **Mean** | **SD** | **95% CI, lower** | **95% CI, upper** | **n** | **Mean** | | **SD** | **95% CI, lower** | **95% CI, upper** | **n** |
| Blank1_TFA | **0.0** | **0.0** | **0.0** | **0.0** | **442** | **0.0** | | **0.0** | **0.0** | **0.0** | **444** |
| Blank2_S1 | **0.4** | **0.3** | **0.0** | **1.1** | **909** | **4.8** | | **2.4** | **0.7** | **9.7** | **666** |
| Blank2_S2 | **0.7** | **0.5** | **0.1** | **1.8** | **300** | **0.0** | | **0.0** | **0.0** | **0.0** | **78** |
| Blank2_P | **2.7** | **2.0** | **0.1** | **7.8** | **593** | **3.7** | | **3.6** | **0.0** | **12.0** | **318** |
| Blank3_TFA | **12.1** | **7.2** | **0.0** | **28.4** | **24** | **13.8** | | **6.9** | **1.4** | **28.6** | **44** |
| Blank4_TFA | 33.1 | 31.1 | 0.0 | 100.0 | 3 | **16.5** | | **7.3** | **0.0** | **30.8** | **20** |

## **Microscopic analysis**

Initially, a total of 15 ochre-rich sediment samples from Klipdrift Shelter (KDS) Howiesons Poort (HP) layers chosen to be representative of those analysed via proteomics (Figure S12) were collected for examination using two different optical microscopes; Nikon Eclipse Ci POL microscope paired with a Nikon DS-Fi3 and Leica M205A microscope paired with a Leica EC3 digital colour camera. Suspected bone chips were isolated and investigated individually. Subsequently, 9 samples, mainly from PBD and PBE, were selected for further examination using scanning electron microscopy (SEM) combined with energy dispersive X-ray spectroscopy (EDS). Suspected bone fragments were identified based on the fragments’ microstructure and their elemental composition.

Bone consists of a mineral component, bone matrix proteins (collagen and non-collagenous proteins), cells and water. The mineralised phase of bone, hydroxyapatite [Ca_10_(PO_4_)_6_(OH)_2_][[24]](https://paperpile.com/c/Vuv3M7/la2gq), comprises the majority of the bone weight (60%-70%) [[25]](https://paperpile.com/c/Vuv3M7/TIKBC) and therefore, calcium and phosphorus together could be considered as a reliable indicator of bone fragments in the sediment samples. Theoretically, hydroxyapatite has a calcium-to-phosphorus molar ratio (Ca/P) of 1.67 and a mass ratio of 2.15. However, due to diagenetic processes [[26]](https://paperpile.com/c/Vuv3M7/AM0gq) and alterations in the bone’s chemical composition and structure, this ratio varies. For example, a chemical equilibrium with the Ca and PO_4_ in the soil solution might result in a higher or lower rate [[27]](https://paperpile.com/c/Vuv3M7/dt92i). Additionally, due to the rough surface of the fragments studied here, the EDS analyses only yielded qualitative to semiquantitative results. Consequently, we would not be able to definitively conclude that a specimen is a bone fragment based solely on this ratio. Thus, our criteria in distinguishing bone fragments are bone-related microstructure as well as the presence of a relatively high concentration of phosphorus (at least above 500 counts). In a few cases (No.3 and 8, Table S12), despite the negligible concentration of P, we still classified these fragments as ‘bone-like’ based on their microstructure features.

Bone microstructure includes any specific structures such as haversian canals, osteocytes and trabeculae. For reference, check [[28,29]](https://paperpile.com/c/Vuv3M7/oVOCp+G2LRw) for their SEM micrographs. Beyond differences in elemental compositions, considering the environmental context of KDS and its proximity to the ocean, these features of bone especially help distinguish it from the porous structure of limestone skeletal remnants of marine organisms such as shellfish which are commonly found in KDS sediments (Figure S13). For references, check [[30–32]](https://paperpile.com/c/Vuv3M7/9Og9r+NZE38+gsjju) for their SEM micrographs of calcareous sands.

Of the total of 15 samples analysed, only 4 contained any bone-like material, and these were represented by just one or two small fragments (<1-3 mm, Figure S14, S15). These fragments were mostly from PBD sub-samples, and none were identified from PBE. Despite the presence of these fragments in only a few of the samples, their low occurrence suggests they were rare in the samples analysed for proteomics.

**Figure S12- The approximate locations of ochre-rich sediment samples collected for proteomics and microscopy analyses.** Samples are represented by symbol and colour, respectively indicating the type of analysis (circle: microscopy; star: proteomics) and the corresponding stratigraphic layer. Adapted image courtesy of Magnus M. Haaland.

**Table S12-** Overview of ochre-rich sediment samples collected for microscopic analysis and the obtained results. These samples date to between 60-64 ka. The Sample ID includes the name of the layer and the quadrant from which the sample was collected.

| **No.** | **Sample ID.** | **Layer** | **Quadrant** | **Excavation year** | **Microscopic observations** |
| --- | --- | --- | --- | --- | --- |
| **1** | PBD_S28b | PBD | S28b | 2011 | One bone-like fragment (<1 mm), showing both bone surface morphology and relative high concentration of P. |
| **2** | PBE_S28b | PBE | S28b | 2011 | No bone-like fragment |
| **3** | PBD_S30a | PBD | S30a | 2012 | Two fragments (<1-2 mm) with bone-like microstructures were identified. Both show high concentrations of Ca, C, and O, but only one displays a comparatively elevated level of P. |
| **4** | PBD_R30d | PBD | R30d | 2012 | One bone-like fragment (<3 mm), showing bone surface morphology. |
| **5** | PBE_R30d | PBE | R30d | 2012 | No bone-like fragment |
| **6** | PBE_R30a | PBE | R30a | 2012 | No bone-like fragment |
| **7** | PBE_R28d | PBE | R28d | 2012 | No bone-like fragment |
| **8** | PCA_R28d | PCA | R28d | 2012 | Two fragments (<1 mm) with bone-like microstructures were identified. Both show high concentrations of Ca, C, and O, while P is nearly absent. |
| **9** | PBD_S30b | PBD | S30b | 2012 | No bone-like fragment detectable by SEM |
| **10** | PBD_Q30d | PBD | Q30d | 2013 | Initial optical microscopic observations suggested no bone-like fragments. |
| **11** | PBD_R30d | PBD | R30d | 2012 | Initial optical microscopic observations suggested no bone-like fragments. |
| **12** | PBA/PBB_R31a | PBA/PBB | R31a | 2013 | Initial optical microscopic observations suggested no bone-like fragments. |
| **13** | PAU_R27b | PAU | R27b | 2013 | Initial optical microscopic observations suggested no bone-like fragments. |
| **14** | PAV_R28a | PAV | R28a | 2012 | Initial optical microscopic observations suggested no bone-like fragments. |
| **15** | PBE_R30d | PBE | R30d | 2012 | Initial optical microscopic observations suggested no bone-like fragments. |

**Figure S13- SEM micrographs and EDS spectrum of a specimen from an ochre-rich sediment sub-sample collected from layer PBD, quadrant S28b.** The elemental composition (CaCO_3_) and the porous microstructure suggest that the specimen is a remnant of limestone skeleton of marine organisms. Images were acquired using a secondary electron (SE) detector at 5 kV and 4 mm working distance. EDS point analyses were performed at 15 kV and 8.5 mm working distance. The EDS data is presented as a graph with X-ray energy (KeV) on the x-axis and the peak intensity (counts) on the Y-axis.

**Figure S14- SEM micrographs and EDS spectrum of a specimen from an ochre-rich sediment sub-sample collected from layer PBD, quadrant S28b.** The presence of relative high P and Ca counts as well as the possible vascular canal of bone suggest that the specimen is a bone fragment. Images were acquired using a secondary electron (SE) detector at 5 kV and 4 mm working distance. EDS point analyses were performed at 15 kV and 8.5 mm working distance. The EDS data is presented as a graph with X-ray energy (KeV) on the x-axis and the peak intensity (counts) on the Y-axis.

**Figure S15- SEM micrographs and EDS spectrum of a specimen from an ochre-rich sediment sub-sample collected from layer PBD, quadrant S30a.** The presence of relatively high P and Ca counts as well as the surface morphology suggest that the specimen is a bone fragment. Images were acquired using a secondary electron (SE) detector at 5 kV and 4 mm working distance. EDS point analyses were performed at 15 kV and 8.5 mm working distance. The EDS data is presented as a graph with X-ray energy (KeV) on the x-axis and the peak intensity (counts) on the Y-axis.

## **Scenarios for the anthropogenic origin of eland protein**

Based on stringent criteria for protein identification and sequence evaluation, the observed molecular damage patterns (such as extensive deamidation and fragmentation), and the archaeological context, it is unlikely that the eland‑derived proteins represent modern contamination. In this study, acid treatment in both extraction protocols resulted in a high number of peptide identifications (Table S4). This indicates that the peptides were strongly associated with the mineral components of the sediment, particularly ochre. Such strong mineral-protein interactions may have contributed to the preservation of proteins over approximately 60 000 years. At the same time, these interactions likely required harsh extraction conditions to be disrupted in order to recover a substantial number of peptides. Although it is beyond the scope of this study to identify the exact mechanisms underlying the survival of eland collagen type I, the iron‑rich nature of ochre may also have played a role. Iron has been suggested to stabilise proteins through processes such as iron‑mediated radical cross‑linking and tissue fixation. However, these hypotheses require dedicated research designed to evaluate all potential factors influencing the preservation of eland proteins. Further discussion of these mechanisms can be found in several papers [[33–35]](https://paperpile.com/c/Vuv3M7/yiWm0+CE73W+gXAo7). If strong mineral binding was indeed crucial for protein survival, it becomes implausible that proteins originating from overlying deposits, even if such leaching occurred, would have been secondarily trapped and preserved within the ochre matrix.

In this section, the proteomics data is examined in the context of the current ethnographic and experimental evidence for ochre use. While the available data does not allow for unambiguous conclusions, we propose the following potential scenarios for the association of eland-derived proteins with the ochre surface feature at Klipdrift Shelter:

**Hide processing:** The hypothesis that ochre was used for hide processing is largely based on the presence of ochre residues on lithic end-scrapers and blades, especially given the abundance and frequency of ochre materials at certain South African sites [[36–39]](https://paperpile.com/c/Vuv3M7/Dahq2+yyKg1+V9mzb+dP1Ie). Several experimental studies have demonstrated that ochre can effectively prevent hide degradation [[40,41]](https://paperpile.com/c/Vuv3M7/PZBDD+KCKdx), and may also aid in mechanical finishing or cleaning [[42]](https://paperpile.com/c/Vuv3M7/Hxcog). Ethnographic evidence from Africa suggests that ochre is typically applied during the later stages of hide treatment, particularly for colouring or ‘loading’ the hides [[40,43]](https://paperpile.com/c/Vuv3M7/iwl6E+PZBDD) and not necessarily during the initial stages of processing [[44]](https://paperpile.com/c/Vuv3M7/P0XEn). To achieve effective colouring, ochre must be finely ground to allow particles to adhere to the skin surface [[44]](https://paperpile.com/c/Vuv3M7/P0XEn). Often these finely processed grains are mixed with liquids or other binding agents to facilitate its attachment to the skin [[44]](https://paperpile.com/c/Vuv3M7/P0XEn).

At KDS, a thin-section sample from layer PBE (Figure S2) includes a small patch of ochre surface containing numerous highly processed, rounded and sub-rounded particles (~1 µm; Figure S4). Larger sub-rounded ochre fragments (~2–5 mm) are also present in the same section and throughout the ochre surface. It is possible that larger grains of ochre were used during hide processing and finer particles were incidentally generated as a byproduct. Proteomic analysis did not detect collagen type III alpha 1 (COL3A1), a protein typically associated with skin or hide tissues. We specifically searched against all available COL3A1 sequences for artiodactyls, as a COL3A1 sequence is not available for eland. However, both COL1A1 and COL1A2 are also present in skin (or hide) and are indeed more abundant than COL3A1 in such tissues [[45]](https://paperpile.com/c/Vuv3M7/Au0CH). Notably, the relative abundance of COL3A1 in skin has been shown to decrease with age [[46–48]](https://paperpile.com/c/Vuv3M7/LhBsP+qM7JC+9S10s), which may potentially favour the detectability of type I collagen over type III collagen in proteomic analyses of archaeological hide samples. Alternatively, the absence of COL3A1 may suggest that the collagen proteins originate from a non-hide source. Due to the limited availability of eland-specific protein sequences in public databases, we cannot currently confirm whether the detected collagen derives from eland skin. A more comprehensive reference database could help elucidate the actual tissue involved in the future.

**Binding medium:** Another potential scenario for the presence of eland-derived proteins associated with ochre powder is that the eland protein was used as a component in an ochre-mixture which was subsequently deposited at the site. The ochre mixture could have been used for a variety of purposes, including use as a paint.

Ethnographic records document the use of fat and blood (of a freshly killed eland) as paint binders [[49]](https://paperpile.com/c/Vuv3M7/aeeTv), sometimes mixed with ochre [[50,51]](https://paperpile.com/c/Vuv3M7/CX9fk+iAGrr). Although scientific experiments have not yet conclusively supported this practice [[52–56]](https://paperpile.com/c/Vuv3M7/4J3ht+BeFsb+RlyDa+aEdyO+zotN8), the incidental incorporation of collagen proteins during binder preparation is a reasonable possibility, especially given that type I collagen is the most abundant structural protein in animal tissues. In addition, we did not detect any blood proteins, such as albumin or haemoglobin, in our samples, even though reference sequences from other Bovidae taxa (e.g. *Bos taurus*) are available in public databases. However, the absence of these proteins does not rule out the possibility that blood was originally present, as blood-derived proteins may simply have degraded beyond detection limits of current analytical approaches. Furthermore, the presence of coarse ochre particles (~2–5 mm) in the ochre surface at KDS does not align with known practices of paint production. Ethnographic accounts and analytical studies of pigments associated with San rock art consistently indicate the use of finely ground ochre for painting purposes [[55,57,58]](https://paperpile.com/c/Vuv3M7/QLGF6+aEdyO+YGVav). This discrepancy may suggest that the association of eland-derived proteins with the ochre surface feature is unlikely to result from paint deposition alone. It is also possible that multiple ochre-related activities contributed to the formation of the feature, and that the coarse particles were introduced through other processes.

Further studies on the micromorphological nature of such ochre deposits, combined with a database enabling the identification of the specific tissue involved may shed light on the behaviours associated with the deposition of this feature.

## **The significance of eland in the lives of hunter-gatherers**

Faunal remains from several southern African archaeological sites indicate that eland was frequently hunted during the Middle Stone Age (MSA) and Later Stone Age (LSA), and therefore formed part of the hunter-gatherers’ diet at the time [[59–61]](https://paperpile.com/c/Vuv3M7/tQsrj+Z2iwm+bGpzT). As a large and docile antelope, the eland was a highly desirable source of food, providing a substantial amount of meat and fat to sustain a large group of people. Beyond its dietary value, the eland held cultural significance in the recent past, as evidenced by the fact that it is the most represented antelope in African rock art. Some of these depictions are argued to be either entirely imaginary, representing shamanic hallucinations, or involving a live animal used in actual rites [[62]](https://paperpile.com/c/Vuv3M7/XVW24). These depictions are often interpreted not merely as eland hunting, but as representing a complex interplay of rituals intricately associated with the eland, its killing and trance dance associated with the dying eland [[63]](https://paperpile.com/c/Vuv3M7/zWTlg). The death of the eland was particularly considered a significant event through which the great potency possessed by the eland was released [[50]](https://paperpile.com/c/Vuv3M7/CX9fk). Shamans sought to harness this power through trance dance performed with rhythmic movements, singing and often the clapping of women [[64]](https://paperpile.com/c/Vuv3M7/91R34). These collective activities likely facilitated shaman’s entry into an altered state of consciousness, or trance. The acquired potency was then directed towards a variety of purposes, such as healing various ailments and game control [[65]](https://paperpile.com/c/Vuv3M7/enj1l). Moreover, based on ethnographic accounts, the San people, indigenous hunter-gatherers in southern Africa, are known to have used a specific type of ochre (*qhang qhang*) as a pigment mixed with the blood of (freshly killed) eland and its fat as binding medium, to prepare San paint for use in rituals [[49–51]](https://paperpile.com/c/Vuv3M7/aeeTv+iAGrr+CX9fk). The careful selection of the pigments and the ritually significant nature of eland blood and fat suggests that San paint, in certain ritual contexts such as those associated with rain-making, was likely intended to be more than a mere substance for image-making, but rather a medium through which spiritual power was believed to be transferred and activated [[50,62,66,67]](https://paperpile.com/c/Vuv3M7/UvwoE+XVW24+BADc1+CX9fk). Eland has also played a central role in several ritual ceremonies, including the boys’ first hunting (first-kill rites) [[68]](https://paperpile.com/c/Vuv3M7/KdKes), the girls’ puberty ceremony, marriage [[68]](https://paperpile.com/c/Vuv3M7/KdKes) and rain-making rites [[49,62,66,67]](https://paperpile.com/c/Vuv3M7/UvwoE+aeeTv+XVW24+BADc1). Thus, the eland’s multifaceted role in both subsistence strategies and rituals highlights its profound significance in the lives of African hunter-gatherers.

Current ethnographic evidence, combined with our proteomics data, does not establish a connection between the belief systems of LSA hunter-gatherers and those of MSA hunter-gatherers, nor determine the extent to which these beliefs might have influenced practical aspects of their lives. However, we do know that some portion of an eland was brought back to Klipdrift Shelter and intermingled with ochre materials in some way. The specific purposes for this action are yet unidentified, but we suggest that the eland remains and its mixture with ochre powder served a multitude of purposes. Given the prominent role of eland in recent southern African symbolic systems, this particular association may hint at early forms of cultural meaning attached to utilitarian practices. However, we caution that such cultural associations cannot be directly projected back to populations living 60 000 years ago.

## **References**

1.	[Henshilwood CS *et al.* 2014 Klipdrift Shelter, southern Cape, South Africa: preliminary report on the Howiesons Poort layers. *J. Archaeol. Sci.* **45**, 284–303. (doi:](http://paperpile.com/b/Vuv3M7/1E02c)[10.1016/j.jas.2014.01.033](http://dx.doi.org/10.1016/j.jas.2014.01.033)[)](http://paperpile.com/b/Vuv3M7/1E02c)

2.	[Velliky EC, MacDonald BL, Haaland MM, Van Niekerk KL, Henshilwood CS. 2026 Ochre and manganese pigments from the Middle Stone Age layers at Klipdrift Shelter. *Archaeol. Anthropol. Sci.* **18**. (doi:](http://paperpile.com/b/Vuv3M7/1QosB)[10.1007/s12520-025-02376-3](http://dx.doi.org/10.1007/s12520-025-02376-3)[)](http://paperpile.com/b/Vuv3M7/1QosB)

3.	[Harvati K, Bauer CC, Grine FE, Benazzi S, Ackermann RR, van Niekerk KL, Henshilwood CS. 2015 A human deciduous molar from the Middle Stone Age (Howiesons Poort) of Klipdrift Shelter, South Africa. *J. Hum. Evol.* **82**, 190–196. (doi:](http://paperpile.com/b/Vuv3M7/T931G)[10.1016/j.jhevol.2015.03.001](http://dx.doi.org/10.1016/j.jhevol.2015.03.001)[)](http://paperpile.com/b/Vuv3M7/T931G)

4.	[Sawafuji R, Cappellini E, Nagaoka T, Fotakis AK, Jersie-Christensen RR, Olsen JV, Hirata K, Ueda S. 2017 Proteomic profiling of archaeological human bone. *R. Soc. Open Sci.* **4**, 161004. (doi:](http://paperpile.com/b/Vuv3M7/cgI7X)[10.1098/rsos.161004](http://dx.doi.org/10.1098/rsos.161004)[)](http://paperpile.com/b/Vuv3M7/cgI7X)

5.	[Tsutaya T, Mackie M, Koenig C, Sato T, Weber A, Kato H, Olsen J, Cappellini E. 2019 Palaeoproteomic identification of breast milk protein residues from the archaeological skeletal remains of a neonatal dog. *Sci. Rep.* **9**, 12841. (doi:](http://paperpile.com/b/Vuv3M7/nAa6p)[10.1038/s41598-019-49183-0](http://dx.doi.org/10.1038/s41598-019-49183-0)[)](http://paperpile.com/b/Vuv3M7/nAa6p)

6.	[Cappellini E *et al.* 2019 Early Pleistocene enamel proteome from Dmanisi resolves Stephanorhinus phylogeny. *Nature* **574**, 103–107. (doi:](http://paperpile.com/b/Vuv3M7/GGUg2)[10.1038/s41586-019-1555-y](http://dx.doi.org/10.1038/s41586-019-1555-y)[)](http://paperpile.com/b/Vuv3M7/GGUg2)

7.	[Taurozzi AJ *et al.* 2024 Deep-time phylogenetic inference by paleoproteomic analysis of dental enamel. *Nat. Protoc.* **19**, 2085–2116. (doi:](http://paperpile.com/b/Vuv3M7/gmx3u)[10.1038/s41596-024-00975-3](http://dx.doi.org/10.1038/s41596-024-00975-3)[)](http://paperpile.com/b/Vuv3M7/gmx3u)

8.	[Rappsilber J, Mann M, Ishihama Y. 2007 Protocol for micro-purification, enrichment, pre-fractionation and storage of peptides for proteomics using StageTips. *Nat. Protoc.* **2**, 1896–1906. (doi:](http://paperpile.com/b/Vuv3M7/4EKEe)[10.1038/nprot.2007.261](http://dx.doi.org/10.1038/nprot.2007.261)[)](http://paperpile.com/b/Vuv3M7/4EKEe)

9.	[Le Meillour L *et al.* 2023 The name of the game: palaeoproteomics and radiocarbon dates further refine the presence and dispersal of caprines in eastern and southern Africa. *R Soc Open Sci* **10**, 231002. (doi:](http://paperpile.com/b/Vuv3M7/JZWkX)[10.1098/rsos.231002](http://dx.doi.org/10.1098/rsos.231002)[)](http://paperpile.com/b/Vuv3M7/JZWkX)

10.	[Janzen A *et al.* 2021 Distinguishing African bovids using Zooarchaeology by Mass Spectrometry (ZooMS): New peptide markers and insights into Iron Age economies in Zambia. *PLoS One* **16**, e0251061. (doi:](http://paperpile.com/b/Vuv3M7/o8PdG)[10.1371/journal.pone.0251061](http://dx.doi.org/10.1371/journal.pone.0251061)[)](http://paperpile.com/b/Vuv3M7/o8PdG)

11.	[Altschul SF, Gish W, Miller W, Myers EW, Lipman DJ. 1990 Basic local alignment search tool. *J. Mol. Biol.* **215**, 403–410. (doi:](http://paperpile.com/b/Vuv3M7/sAMkv)[10.1016/S0022-2836(05)80360-2](http://dx.doi.org/10.1016/S0022-2836(05)80360-2)[)](http://paperpile.com/b/Vuv3M7/sAMkv)

12.	[Kovacs JM, Mant CT, Hodges RS. 2006 Determination of intrinsic hydrophilicity/hydrophobicity of amino acid side chains in peptides in the absence of nearest-neighbor or conformational effects. *Biopolymers* **84**, 283–297. (doi:](http://paperpile.com/b/Vuv3M7/jGp0N)[10.1002/bip.20417](http://dx.doi.org/10.1002/bip.20417)[)](http://paperpile.com/b/Vuv3M7/jGp0N)

13.	[Pacios LF. 2001 Distinct molecular surfaces and hydrophobicity of amino acid residues in proteins. *J. Chem. Inf. Comput. Sci.* **41**, 1427–1435. (doi:](http://paperpile.com/b/Vuv3M7/3cgF6)[10.1021/ci010369n](http://dx.doi.org/10.1021/ci010369n)[)](http://paperpile.com/b/Vuv3M7/3cgF6)

14.	[Xia H *et al.* 2024 Middle and Late Pleistocene Denisovan subsistence at Baishiya Karst Cave. *Nature* **632**, 108–113. (doi:](http://paperpile.com/b/Vuv3M7/0uXk2)[10.1038/s41586-024-07612-9](http://dx.doi.org/10.1038/s41586-024-07612-9)[)](http://paperpile.com/b/Vuv3M7/0uXk2)

15.	[Waterhouse AM, Procter JB, Martin DMA, Clamp M, Barton GJ. 2009 Jalview Version 2—a multiple sequence alignment editor and analysis workbench. *Bioinformatics* **25**, 1189–1191. (doi:](http://paperpile.com/b/Vuv3M7/zwIl0)[10.1093/bioinformatics/btp033](http://dx.doi.org/10.1093/bioinformatics/btp033)[)](http://paperpile.com/b/Vuv3M7/zwIl0)

16.	[Robinson AB, Rudd CJ. 1974 Deamidation of glutaminyl and asparaginyl residues in peptides and proteins. *Current Topics in Cellular Regulation*. , 247–295. (doi:](http://paperpile.com/b/Vuv3M7/vOTkV)[10.1016/b978-0-12-152808-9.50013-4](http://dx.doi.org/10.1016/b978-0-12-152808-9.50013-4)[)](http://paperpile.com/b/Vuv3M7/vOTkV)

17.	[Leo G, Bonaduce I, Andreotti A, Marino G, Pucci P, Colombini MP, Birolo L. 2011 Deamidation at asparagine and glutamine as a major modification upon deterioration/aging of proteinaceous binders in mural paintings. *Anal. Chem.* **83**, 2056–2064. (doi:](http://paperpile.com/b/Vuv3M7/ZM4OT)[10.1021/ac1027275](http://dx.doi.org/10.1021/ac1027275)[)](http://paperpile.com/b/Vuv3M7/ZM4OT)

18.	[Schroeter ER, Cleland TP. 2016 Glutamine deamidation: an indicator of antiquity, or preservational quality? *Rapid Commun. Mass Spectrom.* **30**, 251–255. (doi:](http://paperpile.com/b/Vuv3M7/OqJCN)[10.1002/rcm.7445](http://dx.doi.org/10.1002/rcm.7445)[)](http://paperpile.com/b/Vuv3M7/OqJCN)

19.	[Ramsøe A, van Heekeren V, Ponce P, Fischer R, Barnes I, Speller C, Collins MJ. 2020 DeamiDATE 1.0: Site-specific deamidation as a tool to assess authenticity of members of ancient proteomes. *J. Archaeol. Sci.* **115**, 105080. (doi:](http://paperpile.com/b/Vuv3M7/XqkH3)[10.1016/j.jas.2020.105080](http://dx.doi.org/10.1016/j.jas.2020.105080)[)](http://paperpile.com/b/Vuv3M7/XqkH3)

20.	[Brown S, Kozlikin M, Shunkov M, Derevianko A, Higham T, Douka K, Richter KK. 2021 Examining collagen preservation through glutamine deamidation at Denisova Cave. *J. Archaeol. Sci.* **133**, 105454. (doi:](http://paperpile.com/b/Vuv3M7/aBnb9)[10.1016/j.jas.2021.105454](http://dx.doi.org/10.1016/j.jas.2021.105454)[)](http://paperpile.com/b/Vuv3M7/aBnb9)

21.	[Simpson JP, Penkman KEH, Demarchi B, Koon H, Collins MJ, Thomas-Oates J, Shapiro B, Stark M, Wilson J. 2016 The effects of demineralisation and sampling point variability on the measurement of glutamine deamidation in type I collagen extracted from bone. *J. Archaeol. Sci.* **69**, 29–38. (doi:](http://paperpile.com/b/Vuv3M7/Osal8)[10.1016/j.jas.2016.02.002](http://dx.doi.org/10.1016/j.jas.2016.02.002)[)](http://paperpile.com/b/Vuv3M7/Osal8)

22.	[Tsutaya T. 2025 *rDeamidation*. Zenodo. (doi:](http://paperpile.com/b/Vuv3M7/eV4zl)[10.5281/zenodo.17654645](http://dx.doi.org/10.5281/zenodo.17654645)[)](http://paperpile.com/b/Vuv3M7/eV4zl)

23.	[Mackie M *et al.* 2018 Palaeoproteomic profiling of conservation layers on a 14th century Italian wall painting. *Angew. Chem. Int. Ed Engl.* **57**, 7369–7374. (doi:](http://paperpile.com/b/Vuv3M7/Qgm8t)[10.1002/anie.201713020](http://dx.doi.org/10.1002/anie.201713020)[)](http://paperpile.com/b/Vuv3M7/Qgm8t)

24.	[Bonucci E. 2000 Basic Composition and Structure of Bone. In *Mechanical testing of bone and the bone-implant interface*, pp. 3–21. Boca Raton: CRC Press.](http://paperpile.com/b/Vuv3M7/la2gq)

25.	[Morgan EF, Gerstenfeld LC. 2021 Chapter 2 - The bone organ system: form and function. In *Marcus and Feldman’s Osteoporosis (Fifth Edition)* (eds DW Dempster, JA Cauley, ML Bouxsein, F Cosman), pp. 15–35. Academic Press. (doi:](http://paperpile.com/b/Vuv3M7/TIKBC)[10.1016/B978-0-12-813073-5.00002-2](http://dx.doi.org/10.1016/B978-0-12-813073-5.00002-2)[)](http://paperpile.com/b/Vuv3M7/TIKBC)

26.	[Hedges REM. 2002 Bone diagenesis: an overview of processes. *Archaeometry* **44**, 319–328. (doi:](http://paperpile.com/b/Vuv3M7/AM0gq)[10.1111/1475-4754.00064](http://dx.doi.org/10.1111/1475-4754.00064)[)](http://paperpile.com/b/Vuv3M7/AM0gq)

27.	[White EM, Hannus LA. 1983 Chemical weathering of bone in archaeological soils. *American Antiquity* **48**, 316–322.](http://paperpile.com/b/Vuv3M7/dt92i)

28.	[Ellingham STD, Thompson TJU, Islam M. 2018 Scanning electron microscopy-energy-dispersive X-ray (SEM/EDX): A rapid diagnostic tool to aid the identification of burnt bone and contested cremains. *J. Forensic Sci.* **63**, 504–510. (doi:](http://paperpile.com/b/Vuv3M7/oVOCp)[10.1111/1556-4029.13541](http://dx.doi.org/10.1111/1556-4029.13541)[)](http://paperpile.com/b/Vuv3M7/oVOCp)

29.	[Boyde A, Jones SJ. 1996 Scanning electron microscopy of bone: instrument, specimen, and issues. *Microsc. Res. Tech.* **33**, 92–120. (doi:](http://paperpile.com/b/Vuv3M7/G2LRw)[10.1002/(SICI)1097-0029(19960201)33:2<92::AID-JEMT2>3.0.CO;2-0](http://dx.doi.org/10.1002/(SICI)1097-0029(19960201)33:2%3C92::AID-JEMT2%3E3.0.CO;2-0)[)](http://paperpile.com/b/Vuv3M7/G2LRw)

30.	[Dong K, Jiang K, Ruan W. 2023 The strain rate effects of coral sand at different relative densities and moisture contents. *Materials (Basel)* **16**, 4217. (doi:](http://paperpile.com/b/Vuv3M7/9Og9r)[10.3390/ma16124217](http://dx.doi.org/10.3390/ma16124217)[)](http://paperpile.com/b/Vuv3M7/9Og9r)

31.	[Liu K, Yu R, Shui Z, Yi S, Li X, Ling G, He Y. 2020 Influence of external water introduced by coral sand on autogenous shrinkage and microstructure development of Ultra-High Strength Concrete (UHSC). *Constr. Build. Mater.* **252**, 119111. (doi:](http://paperpile.com/b/Vuv3M7/NZE38)[10.1016/j.conbuildmat.2020.119111](http://dx.doi.org/10.1016/j.conbuildmat.2020.119111)[)](http://paperpile.com/b/Vuv3M7/NZE38)

32.	[Zhao Z, Qiu Y, Wang M. 2019 Effects of strain rate and initial density on the dynamic mechanical behaviour of dry calcareous sand. *Shock Vib.* **2019**. (doi:](http://paperpile.com/b/Vuv3M7/gsjju)[10.1155/2019/3526727](http://dx.doi.org/10.1155/2019/3526727)[)](http://paperpile.com/b/Vuv3M7/gsjju)

33.	[Boatman EM, Goodwin MB, Holman H-YN, Fakra S, Zheng W, Gronsky R, Schweitzer MH. 2019 Mechanisms of soft tissue and protein preservation in Tyrannosaurus rex. *Sci. Rep.* **9**, 15678. (doi:](http://paperpile.com/b/Vuv3M7/yiWm0)[10.1038/s41598-019-51680-1](http://dx.doi.org/10.1038/s41598-019-51680-1)[)](http://paperpile.com/b/Vuv3M7/yiWm0)

34.	[Schweitzer MH, Zheng W, Cleland TP, Goodwin MB, Boatman E, Theil E, Marcus MA, Fakra SC. 2014 A role for iron and oxygen chemistry in preserving soft tissues, cells and molecules from deep time. *Proc. Biol. Sci.* **281**, 20132741. (doi:](http://paperpile.com/b/Vuv3M7/CE73W)[10.1098/rspb.2013.2741](http://dx.doi.org/10.1098/rspb.2013.2741)[)](http://paperpile.com/b/Vuv3M7/CE73W)

35.	[Schweitzer MH, Wittmeyer JL, Horner JR. 2007 Soft tissue and cellular preservation in vertebrate skeletal elements from the Cretaceous to the present. *Proc. Biol. Sci.* **274**, 183–197. (doi:](http://paperpile.com/b/Vuv3M7/gXAo7)[10.1098/rspb.2006.3705](http://dx.doi.org/10.1098/rspb.2006.3705)[)](http://paperpile.com/b/Vuv3M7/gXAo7)

36.	[Klein RG. 1995 Anatomy, behavior, and modern human origins. *J. World Prehist.* **9**, 167–198. (doi:](http://paperpile.com/b/Vuv3M7/Dahq2)[10.1007/bf02221838](http://dx.doi.org/10.1007/bf02221838)[)](http://paperpile.com/b/Vuv3M7/Dahq2)

37.	[Mithen S. 1999 8 symbolism and the supernatural. In *The Evolution of Culture*, pp. 147–170. Edinburgh University Press. (doi:](http://paperpile.com/b/Vuv3M7/yyKg1)[10.1515/9781474467889-010](http://dx.doi.org/10.1515/9781474467889-010)[)](http://paperpile.com/b/Vuv3M7/yyKg1)

38.	[Wadley L. 1993 The Pleistocene Later Stone Age south of the Limpopo River. *J. World Prehist.* **7**, 243–296. (doi:](http://paperpile.com/b/Vuv3M7/V9mzb)[10.1007/bf00974721](http://dx.doi.org/10.1007/bf00974721)[)](http://paperpile.com/b/Vuv3M7/V9mzb)

39.	[Wadley L. 2001 What is cultural modernity? A general view and a south African perspective from Rose cottage cave. *Camb. Archaeol. J.* **11**, 201–221. (doi:](http://paperpile.com/b/Vuv3M7/dP1Ie)[10.1017/s0959774301000117](http://dx.doi.org/10.1017/s0959774301000117)[)](http://paperpile.com/b/Vuv3M7/dP1Ie)

40.	[Rifkin RF. 2011 Assessing the efficacy of red ochre as a prehistoric hide tanning ingredient. *Journal of African Archaeology* **9**, 131–158. (doi:](http://paperpile.com/b/Vuv3M7/PZBDD)[10.3213/2191-5784-10199](http://dx.doi.org/10.3213/2191-5784-10199)[)](http://paperpile.com/b/Vuv3M7/PZBDD)

41.	[Audouin F, Plisson H. 1982 Les ocres et leurs témoins au Paléolithique en France: enquête et expériences sur leur validité archéologique. *Cahiers du Centre de Recherches Préhistoriques Paris* **8**, 33–80.](http://paperpile.com/b/Vuv3M7/KCKdx)

42.	[Salomon H, Casseyas C, Cécile J. 2016 Traitement des peaux à l’ocre : mythes et réalités. *Bulletin de la Société royale belge d’études géologiques et archéologiques «Les Chercheurs de la Wallonie* **52**, 171–190.](http://paperpile.com/b/Vuv3M7/Hxcog)

43.	[Bleek W, Lloyd LC. 2001 *Specimens of bushmen folklore*. Einsiedeln, Switzerland: Daimon Verlag.](http://paperpile.com/b/Vuv3M7/iwl6E)

44.	[Badenhorst S. 2009 An ethnographic and historical overview of hide processing in southern Africa. *Annals of the Transvaal Museum* **46**, 37–43. (doi:](http://paperpile.com/b/Vuv3M7/P0XEn)[10.10520/ejc83689](http://dx.doi.org/10.10520/ejc83689)[)](http://paperpile.com/b/Vuv3M7/P0XEn)

45.	[Ramshaw JAM, Madhan B. 2024 Hides and skins: Unravelling nature’s marvel. In *Emerging Trends in Leather Science and Technology*, pp. 3–46. Singapore: Springer Nature Singapore. (doi:](http://paperpile.com/b/Vuv3M7/Au0CH)[10.1007/978-981-99-9754-1_1](http://dx.doi.org/10.1007/978-981-99-9754-1_1)[)](http://paperpile.com/b/Vuv3M7/Au0CH)

46.	[Mays PK, Bishop JE, Laurent G. 1988 Age-related changes in the proportion of types I and III collagen. *Mech. Ageing Dev.* **45**, 203–212. (doi:](http://paperpile.com/b/Vuv3M7/LhBsP)[10.1016/0047-6374(88)90002-4](http://dx.doi.org/10.1016/0047-6374(88)90002-4)[)](http://paperpile.com/b/Vuv3M7/LhBsP)

47.	[Reilly DM, Lozano J. 2021 Skin collagen through the lifestages: Importance for skin health and beauty. *Plastic and Aesthetic Research* **8**, N–A.](http://paperpile.com/b/Vuv3M7/qM7JC)

48.	[Smith LT, Holbrook KA, Madri JA. 1986 Collagen types I, III, and V in human embryonic and fetal skin. *Am. J. Anat.* **175**, 507–521. (doi:](http://paperpile.com/b/Vuv3M7/9S10s)[10.1002/aja.1001750409](http://dx.doi.org/10.1002/aja.1001750409)[)](http://paperpile.com/b/Vuv3M7/9S10s)

49.	[How MW. 1962 *The mountain bushmen of Basutoland*. Pretoria: J L Van Schaik, Ltd. See](http://paperpile.com/b/Vuv3M7/aeeTv) <https://cir.nii.ac.jp/crid/1130282270898640512>[.](http://paperpile.com/b/Vuv3M7/aeeTv)

50.	[Lewis-Williams D. 2019 Patterns of Participation. In *Image-makers: The social context of a Hunter-gatherer ritual*, pp. 59–81. Cambridge, England: Cambridge University Press. (doi:](http://paperpile.com/b/Vuv3M7/CX9fk)[10.1017/9781108632829](http://dx.doi.org/10.1017/9781108632829)[)](http://paperpile.com/b/Vuv3M7/CX9fk)

51.	[Mitchell PJ. 2006/2007 Remembering the Mountain Bushmen: observations of nineteenth century huntergatherers in Lesotho as recorded by Victor Ellenberger. *Southern African Field Archaeology* , 3–10.](http://paperpile.com/b/Vuv3M7/iAGrr)

52.	[Rudner I. 1983 Paints of the Khoisan rock artists. *Goodwin Series* **4**, 14. (doi:](http://paperpile.com/b/Vuv3M7/4J3ht)[10.2307/3858097](http://dx.doi.org/10.2307/3858097)[)](http://paperpile.com/b/Vuv3M7/4J3ht)

53.	[Prinsloo LC, Barnard W, Meiklejohn I, Hall K. 2008 The first Raman spectroscopic study of San rock art in the Ukhahlamba Drakensberg Park, South Africa. *J. Raman Spectrosc.* **39**, 646–654. (doi:](http://paperpile.com/b/Vuv3M7/BeFsb)[10.1002/jrs.1901](http://dx.doi.org/10.1002/jrs.1901)[)](http://paperpile.com/b/Vuv3M7/BeFsb)

54.	[Tournié A, Prinsloo LC, Paris C, Colomban P, Smith B. 2011 The first in situ Raman spectroscopic study of San rock art in South Africa: procedures and preliminary results: Raman spectroscopic study of San rock art in South Africa. *J. Raman Spectrosc.* **42**, 399–406. (doi:](http://paperpile.com/b/Vuv3M7/RlyDa)[10.1002/jrs.2682](http://dx.doi.org/10.1002/jrs.2682)[)](http://paperpile.com/b/Vuv3M7/RlyDa)

55.	[Hœrlé S, Pearce DG, Bertrand L, Sandt CH, Menu M. 2016 Imaging the layered fabric of paints from nomansland rock art (South Africa): Rock art materials from the nomansland, South Africa. *Archaeometry* **58**, 182–199. (doi:](http://paperpile.com/b/Vuv3M7/aEdyO)[10.1111/arcm.12235](http://dx.doi.org/10.1111/arcm.12235)[)](http://paperpile.com/b/Vuv3M7/aEdyO)

56.	[Williamson BS. 2000 Direct testing of rock painting pigments for traces of haemoglobin at Rose cottage cave, South Africa. *J. Archaeol. Sci.* **27**, 755–762. (doi:](http://paperpile.com/b/Vuv3M7/zotN8)[10.1006/jasc.1999.0489](http://dx.doi.org/10.1006/jasc.1999.0489)[)](http://paperpile.com/b/Vuv3M7/zotN8)

57.	[Bonneau A, Pearce DG, Mitchell PJ, Didier L, Eoin LN, Higham TFG, Lamothe M, Arthur C. 2022 Characterization and dating of San rock art in the Metolong catchment, Lesotho: A preliminary investigation of technological and stylistic changes. *Quat. Int.* **611-612**, 177–189. (doi:](http://paperpile.com/b/Vuv3M7/QLGF6)[10.1016/j.quaint.2021.03.014](http://dx.doi.org/10.1016/j.quaint.2021.03.014)[)](http://paperpile.com/b/Vuv3M7/QLGF6)

58.	[Bonneau A, Pearce DG, Pollard AM. 2012 A multi-technique characterization and provenance study of the pigments used in San rock art, South Africa. *J. Archaeol. Sci.* **39**, 287–294. (doi:](http://paperpile.com/b/Vuv3M7/YGVav)[10.1016/j.jas.2011.09.011](http://dx.doi.org/10.1016/j.jas.2011.09.011)[)](http://paperpile.com/b/Vuv3M7/YGVav)

59.	[Klein RG, Cruz-Uribe K. 1996 Exploitation of large bovids and seals at Middle and Later Stone Age sites in South Africa. *J. Hum. Evol.* **31**, 315–334. (doi:](http://paperpile.com/b/Vuv3M7/tQsrj)[10.1006/jhev.1996.0064](http://dx.doi.org/10.1006/jhev.1996.0064)[)](http://paperpile.com/b/Vuv3M7/tQsrj)

60.	[Faith JT. 2008 Eland, buffalo, and wild pigs: were Middle Stone Age humans ineffective hunters? *J. Hum. Evol.* **55**, 24–36. (doi:](http://paperpile.com/b/Vuv3M7/Z2iwm)[10.1016/j.jhevol.2007.11.005](http://dx.doi.org/10.1016/j.jhevol.2007.11.005)[)](http://paperpile.com/b/Vuv3M7/Z2iwm)

61.	[Dusseldorp GL. 2010 Prey choice during the South African Middle Stone Age: Avoiding dangerous prey or maximising returns? *Afr. Archaeol. Rev.* **27**, 107–133. (doi:](http://paperpile.com/b/Vuv3M7/bGpzT)[10.1007/s10437-010-9072-2](http://dx.doi.org/10.1007/s10437-010-9072-2)[)](http://paperpile.com/b/Vuv3M7/bGpzT)

62.	[Jolly P. 2021 Rock paintings which may depict live eland being used in San rites. *Southern African Humanities* **34**, 111–136.](http://paperpile.com/b/Vuv3M7/XVW24)

63.	[Lewis-Williams JD. 1983 *The rock art of southern Africa*. Cambridge, England: Cambridge University Press.](http://paperpile.com/b/Vuv3M7/zWTlg)

64.	[Lewis-Williams JD. 1987 A dream of eland: An unexplored component of San shamanism and rock art. *World Archaeol.* **19**, 165–177. (doi:](http://paperpile.com/b/Vuv3M7/91R34)[10.1080/00438243.1987.9980032](http://dx.doi.org/10.1080/00438243.1987.9980032)[)](http://paperpile.com/b/Vuv3M7/91R34)

65.	[Lewis-Williams JD. 1992 Ethnographic evidence relating to ‘trance’ and ‘shamans’ among northern and southern bushmen. *S. Afr. Archaeol. Bull.* **47**, 56. (doi:](http://paperpile.com/b/Vuv3M7/enj1l)[10.2307/3888993](http://dx.doi.org/10.2307/3888993)[)](http://paperpile.com/b/Vuv3M7/enj1l)

66.	[Jolly P. 1986 A first generation descendant of the Transkei San. *S. Afr. Archaeol. Bull.* **41**, 6. (doi:](http://paperpile.com/b/Vuv3M7/UvwoE)[10.2307/3887710](http://dx.doi.org/10.2307/3887710)[)](http://paperpile.com/b/Vuv3M7/UvwoE)

67.	[Lewis-Williams JD. 1981 *Believing and seeing: Symbolic meanings in southern San rock paintings*. Academic Press London.](http://paperpile.com/b/Vuv3M7/BADc1)

68.	[Lewis-Williams JD, Biesele M. 1978 Eland hunting rituals among northern and southern San groups: Striking similarities. *Africa* **48**, 117–134. (doi:](http://paperpile.com/b/Vuv3M7/KdKes)[10.2307/1158603](http://dx.doi.org/10.2307/1158603)[)](http://paperpile.com/b/Vuv3M7/KdKes)
